# Distributions, *ex situ* conservation priorities, and genetic resource potential of crop wild relatives of sweetpotato [*Ipomoea batatas* (L.) Lam., *I*. series *Batatas*]

**DOI:** 10.3389/fpls.2015.00251

**Published:** 2015-04-21

**Authors:** Colin K. Khoury, Bettina Heider, Nora P. Castañeda-Álvarez, Harold A. Achicanoy, Chrystian C. Sosa, Richard E. Miller, Robert W. Scotland, John R. I. Wood, Genoveva Rossel, Lauren A. Eserman, Robert L. Jarret, G. C. Yencho, Vivian Bernau, Henry Juarez, Steven Sotelo, Stef de Haan, Paul C. Struik

**Affiliations:** ^1^International Center for Tropical Agriculture, CaliColombia; ^2^Centre for Crop Systems Analysis, Wageningen University, WageningenNetherlands; ^3^International Potato Center, CGIAR Research Program on Roots, Tubers and Bananas, LimaPeru; ^4^School of Biosciences, University of Birmingham, BirminghamUK; ^5^Department of Biological Sciences, Southeastern Louisiana University, Hammond, LAUSA; ^6^Department of Plant Sciences, University of Oxford, OxfordUK; ^7^Department of Plant Biology, University of Georgia, Athens, GAUSA; ^8^Plant Genetic Resources Conservation Unit, United States Department of Agriculture – Agricultural Research Service, Griffin, GAUSA; ^9^Department of Horticultural Science, North Carolina State University, Raleigh, NCUSA; ^10^Department of Horticulture and Crop Science, The Ohio State University, Columbus, OHUSA

**Keywords:** crop diversity, crop improvement, crop wild relatives, food security, gap analysis, plant genetic resources

## Abstract

Crop wild relatives of sweetpotato [*Ipomoea batatas* (L.) Lam., *I.* series *Batatas*] have the potential to contribute to breeding objectives for this important root crop. Uncertainty in regard to species boundaries and their phylogenetic relationships, the limited availability of germplasm with which to perform crosses, and the difficulty of introgression of genes from wild species has constrained their utilization. Here, we compile geographic occurrence data on relevant sweetpotato wild relatives and produce potential distribution models for the species. We then assess the comprehensiveness of *ex situ* germplasm collections, contextualize these results with research and breeding priorities, and use ecogeographic information to identify species with the potential to contribute desirable agronomic traits. The fourteen species that are considered the closest wild relatives of sweetpotato generally occur from the central United States to Argentina, with richness concentrated in Mesoamerica and in the extreme Southeastern United States. Currently designated species differ among themselves and in comparison to the crop in their adaptations to temperature, precipitation, and edaphic characteristics and most species also show considerable intraspecific variation. With 79% of species identified as high priority for further collecting, we find that these crop genetic resources are highly under-represented in *ex situ* conservation systems and thus their availability to breeders and researchers is inadequate. We prioritize taxa and specific geographic locations for further collecting in order to improve the completeness of germplasm collections. In concert with enhanced conservation of sweetpotato wild relatives, further taxonomic research, characterization and evaluation of germplasm, and improving the techniques to overcome barriers to introgression with wild species are needed in order to mobilize these genetic resources for crop breeding.

## Introduction

Sweetpotato [*Ipomoea batatas* (L.) Lam.] counts among the world’s most important root crops, grown on at least eight million hectares in 114 countries worldwide, with particular significance to food supplies in the tropics and subtropics of East and Southeast Asia, and Sub-Saharan Africa. The success of the crop in these regions is due to its adaptability to a wide range of agro-ecological conditions, ease of propagation from cuttings, low input cultivation requirements, and high productivity as well as nutritive value (Woolfe, 1992). The crop can be cultivated from humid to semi-arid conditions, from sea level to 3000 m.a.s.l. (Huaman, 1987), and can translocate photosynthetic products to the storage roots throughout the growing season, thereby mitigating negative effects of temporary adverse conditions (Kays, 1985). Sweetpotato produces among the highest amounts of edible energy per hectare of all major food crops (De Vries et al., 1967), and is an important source of vitamin A and C, calcium, iron, and a number of essential amino acids (Kays and Kays, 1998; Tumwegamire et al., 2011). In addition to human consumption of the storage roots and young leaves, the crop is utilized for animal feed, fuel, and starch production.

In Sub-Saharan Africa, sweetpotato is predominantly cultivated in small plots characterized by low fertility and drought-prone soils, producing relatively good yields with low inputs and minimal labor costs. The crop has recently become the focus of targeted bio-fortification for enhanced vitamin A. Orange-fleshed varieties have been bred with 50-fold more β-carotene than standard varieties and these newly released varieties rank first among roots and tubers in Sub-Saharan Africa for their nutritional quality (Low et al., 2007; Hotz et al., 2012). Given its adaptability, low-external input requirements, nutritional quality, and improvement potential, it is not surprising that sweetpotato has become a priority in crop based strategies for enhancing food security in the tropics (Pfeiffer and McClafferty, 2007; Bill and Melinda Gates Foundation, 2011; Bouis and Islam, 2012).

The full potential of sweetpotato is far from realized, with particularly large yield gaps (ca. 20 t ha^-1^) remaining across rain-fed Sub-Saharan Africa due to a range of biotic and abiotic constraints, especially sweetpotato virus disease (SPVD) and sweetpotato weevils (SPW), as well as susceptibility to drought (Sutherland, 1986; Valverde et al., 2007; Ngailo et al., 2013; Kivuva et al., 2015a,b). SPVD is a severe constraint on the continent, caused by the synergistic interaction of two viruses transmitted by whiteflies and aphids, causing yield losses of up to 98% under severe infections (Ngailo et al., 2013). SPW (*Cylas* spp.) are the most devastating insect pests of the crop. *Cylas formicarius elegantulus* Summers, *C. puncticollis* Boheman and *C. brunneus* Fabricius can cause yield losses of between 67 and 100% in Sub-Saharan Africa (Smit, 1997). The concealed feeding behavior, oviposition, and larval development of the weevils in the storage roots make their control very difficult, necessitating the development of improved management options, in particular via enhanced genetic resistance. Drought, and the compounding effect of increasing heat on drought, is a rising concern particularly in regions undergoing significant climatic change, both due to its direct effect on productivity (Low et al., 2009; Schafleitner et al., 2010) as well as to its association with increased severity of damage from SPW and SPVD (Munyiza et al., 2007; Mwololo et al., 2007). Lack of drought tolerance in high β-carotene sweetpotato varieties has led to constraints in their adoption (Mwanga and Ssemakula, 2011).

Crop wild relatives (CWR) are increasingly being recognized for their potential to contribute valuable traits to breeding programs (Feuillet et al., 2008; Guarino and Lobell, 2011; Dempewolf et al., 2014). CWR have provided breeders with genes for pest and disease resistance, abiotic stress tolerance, and quality traits in an ever increasing number of food crops, such as banana, barley, bean, cassava, chickpea, maize, lettuce, oat, potato, rice, sugarcane, sunflower, tomato, and wheat, among others (Xiao et al., 1996; Hajjar and Hodgkin, 2007; McCouch et al., 2007; Khoury et al., 2010). Yet despite the successful history of contribution to the improvement of major crops, systematic approaches to the use of CWR in the breeding programs of a number of important staples, including sweetpotato, remain underdeveloped.

The morning glory tribe Ipomoeeae contains ~650–900 species and includes the genus *Ipomoea* and nine other related genera (Wilkin, 1999; Mabberley, 2008). Although many genera, subgenera, and sections of the Ipomoeeae are not monophyletic in phylogenetic analyses, *Ipomoea* series *Batatas* (Choisy) D. F. Austin, which contains sweetpotato and 14 closely related CWR (Austin, 1978; McDonald and Austin, 1990), does form a monophyletic lineage (Miller et al., 1999; McDonald et al., 2011; Eserman et al., 2014). These species include wild *I. batatas* (L.) Lam. [including *I. batatas* var. *apiculata* (Martens and Galeotti) McDonald and Austin], *Ipomoea cordatotriloba* Dennstedt, *Ipomoea cynanchifolia* Meisn., *Ipomoea grandiflora* (Dammer) O’Donell, *Ipomoea lacunosa* L., *Ipomoea leucantha* Jacquin, *Ipomoea littoralis* Blume, *Ipomoea ramosissima* (Poir.) Choisy, *Ipomoea splendor-sylvae* House, *Ipomoea tabascana* McDonald and Austin, *Ipomoea tenuissima* Choisy, *Ipomoea tiliacea* (Willd.) Choisy in D. C., *Ipomoea trifida* (H. B. K.) G. Don, and *Ipomoea triloba* L.

Many sweetpotato CWR can be hybridized with the crop through controlled pollinations, somatic cell, and/or ovule culture techniques (Diaz et al., 1996). Crosses involving *I*. *tabascana*, *I*. *trifida*, *I*. *triloba*, *I*. *littoralis*, *I*. *grandifolia*, *I*. *lacunosa*, *I*. *leucantha*, and wild *I*. *batatas* in particular have resulted in relatively viable progeny (Nimmakayala et al., 2011). The wild conspecific as well as *I*. *trifida* have been documented for their contribution to increases in protein and starch content, and nematode and SPW resistance (Iwanaga, 1988; Shiotani et al., 1994), although there is uncertainty for some material as to whether they may actually have been feral forms of the cultivar (Nimmakayala et al., 2011). Species that have been explored for potential traits of use in crop improvement include *I*. *trifida* and *I*. *littoralis* for yield and SPW, scab [*Elsinoë batatas* (Saw.) Viegas et Jenkins], and black rot disease (*Ceratocystis fimbriata* Ell. et Halst.) resistance; *I*. *grandifolia* for sweetpotato stem nematode and SPVD resistance; and *I*. *triloba* for drought tolerance, root rot resistance, and foliar fungal disease resistances (Iwanaga, 1988; Jarret et al., 1992; Komaki, 2004; Zhang and Liu, 2005; Nimmakayala et al., 2011). Challenges in the creation of viable progeny between the CWR and the cultivated species are not insignificant, though, due to differences in ploidy and interspecific incompatibility (Martin, 1970, 1982; Teramura, 1979; Lu and Li, 1992; Shiotani et al., 1994; Diaz et al., 1996; Komaki, 2004; Nimmakayala et al., 2011).

A lack of basic knowledge about boundaries between species within *I*. series *Batatas* and a dearth of diagnostic characters enabling differentiation of taxa – to facilitate reliable and accurate species identification – is a fundamental stumbling block constraining the utilization of sweetpotato CWR (Austin, 1978, 1988; Jarret et al., 1992; Diaz et al., 1996; McDonald et al., 2011; Nimmakayala et al., 2011; Eserman et al., 2014). Needed studies have been delayed in part due to the absence of plant materials for research, in particular the availability of specimens with flowers and ripe fruits. Studies that have been performed have often been based upon limited sampling (e.g., single accessions for *I. littoralis* and *I*. *tabascana*).

Many unanswered questions regarding the relationships of CWR to sweetpotato potentially impact the efficiency of breeding strategies for the crop. For example, do species such as *I. tabascana* and *I*. *tenuissima* represent distinct taxa (hybrid or otherwise) or rather rare variants of the crop? What is the range and genetic diversity present in truly wild forms of the crop conspecific, compared to feral escapees? How accurate are the classifications of species with highly disjunct distributions (e.g., *I*. *cordatotriloba*)? Are there as yet unrecognized cryptic species within *I*. series *Batatas* [e.g., *I*. ‘*austinii*’ (Duncan and Rausher, 2013)]? What are the lineages and genetic resources potential of purported hybrid species (i.e., *I*. *leucantha* and *I*. *grandifolia*)? What are the geographic locations of new variation being generated through hybridization among sweetpotato CWR?

The investigation, conservation, and availability of genetic resources of sweetpotato provide a foundation for the crop’s long term viability and for its potential for improvement. To contribute to these objectives, we analyzed the comprehensiveness of *ex situ* conservation of sweetpotato CWR through a series of questions: (a) what constitutes a potentially useful wild relative of sweetpotato?, (b) where are these species encountered?, (c) what is the state of conservation and availability of these species to researchers, and what are the highest taxonomic and ecogeographic priorities for further collecting? And finally, (d) what traits do sweetpotato CWR possess that may be valuable to crop improvement?

## Materials and Methods

### Identification of Target CWR Species and Occurrence Data Compilation

The CWR of sweetpotato analyzed in this study were selected based upon recent and historical taxonomic and molecular phylogenetic research (Austin, 1978, 1997; Austin and Huamán, 1996; Miller et al., 1999; McDonald et al., 2011; Germplasm Resources Information Network [GRIN], 2013; Eserman et al., 2014), identifying those wild species with a relatively close phylogenetic relationship to the crop (i.e., members of *I*. series *Batatas*). We included all 14 wild species comprising the series in the analysis.

Domesticated sweetpotato *I*. *batatas* (6x) has been proposed as originating from interspecific hybridization involving *I*. *trifida* (2x, 4x, 6x), *I*. *littoralis* (2x), and/or *I*. *leucantha* (2x) (Nishiyama, 1971, 1982; Austin, 1988). The species most closely related to the crop have been posited to be *I. trifida* followed by *I*. *tabascana* (4x) (Srisuwan et al., 2006; Nimmakayala et al., 2011). Following the genepool concept of Harlan and de Wet (1971) and due to ploidy incompatibility with the cultivated species, the putative closest related taxa to sweetpotato are placed in the secondary genepool: wild forms of *I*. *batatas* (4x), *I. trifida*, *I. littoralis*, and *I*. *tabascana* (Jarret et al., 1992; Jarret and Austin, 1994; Rajapakse et al., 2004; Germplasm Resources Information Network [GRIN], 2013). Species classified as tertiary wild relatives include: *I*. *cordatotriloba* (syn. *Ipomoea trichocarpa* Elliott), *I*. *cynanchifolia*, *I*. *grandifolia*, *I*. *lacunosa*, *I. leucantha*, *I*. *ramosissima*, *I*. *splendor-sylvae* (syn. *Ipomoea umbraticola* House), *I*. *tenuissima, I*. *tiliacea*, and *I*. *triloba* (Jarret and Austin, 1994; Huang et al., 2002; Rajapakse et al., 2004; Germplasm Resources Information Network [GRIN], 2013).

Occurrence records for these species were acquired from online biodiversity, herbaria, and germplasm databases; through communications with herbaria and genebank managers, and other crop researchers; and via direct recording of provenance data during visits to selected herbaria (Supplementary Table S1). Germplasm data were obtained from repositories that provide straightforward access to genetic resources and associated data to the global research community through online information systems. The occurrence data were then compiled in a standardized format, nomenclature was checked against Germplasm Resources Information Network [GRIN] Taxonomy for Plants (2013) and The Plant List (2010), and duplicate records were eliminated. Existing coordinates were cross-checked to country and being on land (Hijmans et al., 1999), and records with locality information but no coordinates were geo-referenced using the Google Maps Geocoder (2013) v.3 application programming interface. Occurrence data were mapped, iteratively evaluated for correctness, and further processed in order to form a final dataset of improved taxonomic and spatial accuracy.

Challenges in using and in improving the large quantities of occurrence data now available from online resources such as the Global Biodiversity Information Facility (GBIF) have been noted (Gaiji et al., 2013), including geographic and nomenclatural data quality and the slow speed with which aggregated datasets are updated (Mesibov, 2013; Otegui et al., 2013; Hjarding et al., 2014). In addition, particular caution must be applied to the occurrence records used in the current paper as ongoing work (unpublished data) indicates that many *Ipomoea* occurrence records in such online databases are identified as synonyms, excluded or invalid names, and that many valid names were applied to specimens well outside of species known ranges. We have identified some of these obvious errors but until all specimen records are correctly identified and checked against an accurate taxonomy these data must be treated with caution.

A total of 5,614 occurrence records for the 14 taxa were included in potential distribution modeling and/or in the conservation analysis, including 749 germplasm records sourced from four genebanks, and 4,865 herbarium and other occurrence reference records sourced from 42 providers. Records per species ranged from eight (*I. tabascana*) to 1,409 (*I. trifida*). Of these, 3,650 records containing unique cross-checked coordinates were used to model species potential distributions and to locate the original collecting site of existing germplasm accessions.

### Species Potential Distribution Modeling

A potential distribution model for each species was calculated using the maximum entropy (Maxent) algorithm (Phillips et al., 2006), with a set of ecogeographic variables and unique species presence records as inputs. We chose Maxent due to its extensive application in predicting species distributions (Elith et al., 2006; Phillips and Dudik, 2008; Costa et al., 2010), including those for wild relatives (Ramírez-Villegas et al., 2010; Conolly et al., 2012; Khoury et al., 2015). We performed modeling at a resolution of 2.5 arc-minutes (~5 km^2^ cell size at the equator), employing 10,000 background points for model training over the combined distributional range of the sweetpotato CWR. Ecogeographic inputs included altitude and 19 bioclimatic variables from the WorldClim database (Hijmans et al., 2005), and seven major edaphic drivers of plant species distributions with consistent data coverage throughout the range of the sweetpotato CWR species, obtained from ISRIC-World Soil Information (Hengl et al., 2014; Supplementary Table S2). For the edaphic variables we calculated a weighted mean across 0–5, 5–15, 15–30, 30–60, and 60–100 cm soil depth values in order to derive a single data value for 0–100 cm. We then resampled the 30 arc-seconds resolution data to form 2.5 arc-minutes inputs aligned with the WorldClim datasets.

Potential distribution models were produced by calculating the mean of replicates (*k* = 5), and clipped by measuring the shortest distance between the receiver operating characteristic curve (ROC-curve) and the top-left corner of the plot (Liu et al., 2005). Models were constrained per species by a native range defined at the country level as given in GRIN Taxonomy for Plants (Germplasm Resources Information Network [GRIN], 2013), in order to focus prioritization recommendations on those regions with populations exhibiting long-term adaptations to local ecogeographic conditions. We further cross-validated and refined occurrence data based upon our knowledge of native distributions, constraining localities for wild *I. batatas* to Mexico south to Peru, and not in the Caribbean; *I. leucantha* to the USA and Mexico, and *I. littoralis* to points within 100 km of the ocean.

In order to derive robust distribution models for each species, we analyzed Maxent results across three groups of ecogeographic inputs: (a) the full set of 19 bioclimatic variables (Ramírez-Villegas et al., 2010); (b) the bioclimatic variables, altitude, and the additional set of seven edaphic variables, totaling 27 input variables; and (c) a species-specific derivation of the most important drivers of distribution based upon presence data, further refined by removing highly correlated variables. For the ecogeographic variables in the species-specific subset method, we utilized a non-linear iterative partial least squares (NIPALS) algorithm to perform a principal-component analysis (PCA), as NIPALS has the potential to handle data arrays in which the number of observations is less than the number of input variables. We then identified those variables with the greatest contribution (>0.7 or < -0.7) to the first two principal components per species. Finally, we used a variance inflation factor (VIF) to identify the variables with a low degree of collinearity (see Supplementary Table S3 for a list of variables identified per species). A calibrated area under the ROC curve (cAUC) was obtained to assess the predictive performance of each model (Hijmans, 2012; Khoury et al., 2015).

The three modeling methods were compared against null models, and the species-specific subset variables method showed the least overall spatial sorting bias among methods (Spearman’s *p* for the 19 variables was 0.65, for 27 variables it was 0.72, and for the subset method it was 0.25), although the differences in median AUC distributions across species for each method were not significant (*p* = 0.095) through a Kruskal–Wallis non-parametric analysis of variance test. Maxent models were subjected to a four-fold assessment process including: (a) the fivefold average area under the ROC curve of test data (ATAUC), b) the standard deviation of the test AUC of the five different folds (STAUC), (c) the proportion of the potential distribution coverage with SD above 0.15 (ASD15), and (d) the cAUC value. Models with ATAUC above 0.7, STAUC below 0.15, ASD15 below 10%, and cAUC exceeding 0.40 were considered accurate and stable (Ramírez-Villegas et al., 2010; Khoury et al., 2015).

The potential distribution models of the sweetpotato CWR generally performed well in regard to the modeling assessment process. Four species demonstrated low cAUC values and one of these an elevated ASD15 value, indicating greater uncertainty in the models (Supplementary Table S3). Species-specific subset model outputs for taxa with relatively few distinct occurrence points (<20; *I*. *tabascana*, *I*. *tenuissima*, and *I. cynanchifolia*) lacked sufficient discriminatory power, leading to highly inflated spatial models in comparison to recorded distributions. Potential distribution models for these species were resolved by deriving an ensemble (i.e., overlap) of the three input variation methods, verified by researchers knowledgeable in the distribution of the species as more accurately representing the true known distributions. Potential distribution models based upon the species-specific subset variables method were therefore utilized in subsequent analyses for all species with adequate distinct occurrence points (>20). The ensemble method was used for the three species with limited occurrence data.

### Analysis of Current *Ex Situ* Conservation and Further Collecting Needs for CWR

We adapted a gap analysis methodology proposed by Ramírez-Villegas et al. (2010), combining three metrics used to assess the urgency of further collecting in order to fill gaps in *ex situ* conservation of CWR. The total sample representation of each species in genebank collections was estimated via a sampling representativeness score (SRS), calculated as the number of germplasm samples (G) divided by the total number of samples [G + herbarium samples (H); i.e., all other records aside from available genebank accessions].

The sufficiency of geographic coverage of germplasm collections of each species was estimated through a geographic representativeness score (GRS), calculated as the share of the combined total area of circular buffers of 50 km (CA50) placed around existing germplasm collection points compared with the overall potential geographic distribution of the species.

The comprehensiveness of ecological coverage of germplasm collections of each species was estimated through an ecological representativeness score (ERS), calculated by estimating the distinct ecosystem classifications (Olson et al., 2001) represented in the CA50 of existing germplasm collection points compared with the diversity of ecosystems in which the overall potential geographic distribution model of the species occurs.

A final priority score (FPS) for further collecting for *ex situ* conservation was assigned to each species by averaging the three gap analysis metrics (SRS, GRS, and ERS). FPS scores were further classified into four categories of urgency for collecting: high priority species (HPS) for taxa whose 0 < FPS ≤ 2.5 or when no germplasm accessions are currently conserved, medium priority species (MPS) when 2.5 < FPS ≤ 5, low priority species (LPS) when 5 < FPS ≤ 7.5, and ‘no further collecting recommended’ (NFCR) when 7.5 < FPS ≤ 10. We produced collecting priorities maps for all species, displaying the geographic areas that have not yet been collected from within the potential distributions of taxa.

The ecogeographic data preparation, species distribution modeling, and gap analysis were written and performed in R v2.15.1 (R Core Team, 2013), utilizing packages maptools (Bivand and Lewin-Koh, 2014), rgdal (Bivand et al., 2014), SDMTools (van der Wal et al., 2014), raster (Hijmans, 2014), sp (Pebesma and Bivand, 2005; Bivand et al., 2013), dismo (Hijmans et al., 2013), and plsdepot (Sanchez, 2012). Resulting spatial files were mapped in ArcMap v.10 (ESRI, 2011). Collecting priorities spatial files were analyzed using the Zonal Statistics tool in ArcMap to list the countries prioritized for further collecting for *ex situ* conservation.

### Expert Evaluation of Conservation Assessment Results

In order to validate and/or expose deficiencies in our findings, we subjected the gap analysis numerical and spatial results to an evaluation performed by seven crop experts with experience in the systematics, distribution, and/or conservation status of CWR of sweetpotato: Richard E. Miller, Southeastern Louisiana University; Robert W. Scotland and John R. I. Wood, University of Oxford; Genoveva Rossel, International Potato Center; Lauren A. Eserman, University of Georgia; Robert L. Jarret, USDA – ARS Griffin; and G. Craig Yencho, North Carolina State University. These experts were first asked to provide an evaluation of the sufficiency of germplasm collections per species based only upon their knowledge of the total number of accessions conserved, and geographic as well as environmental gaps. Such an assessment [comparable expert priority score (EPS)] was considered directly comparable to the FPS of the gap analysis results.

A second evaluation score (contextual EPS) based on the entirety of expert knowledge, including additional factors such as threats to species *in situ* and prioritization by usefulness in crop breeding, was collected in order to provide additional information to collecting prioritization efforts. In both cases, an EPS between 0 and 10, aligned with the gap analysis results prioritization scale, was requested. After these steps, experts were shown the gap analysis data and asked to comment on assessed quantitative results, occurrence data, potential distribution models, and maps of collecting priorities. Following these contributions by experts, input occurrence data were further refined by eliminating clearly incorrect points and adjusting country-level native areas, and the potential distribution modeling and gap analyses were re-run using the refined datasets in order to improve the quantitative and spatial outputs. Expert evaluation metrics displayed in the results pertain to a final evaluation of improved gap analysis outputs, performed by five of the experts.

A multiple factor analysis was used in order to compare the various forms of expert evaluation inputs (i.e., comparable expert priority score, contextual expert priority score, evaluation of gap analysis results score, evaluation of occurrence data, evaluation of potential species distribution models, and evaluation of collecting priorities map) with the gap analysis results. An expert evaluation index was created, which estimated the degree of accord between all experts and the gap analysis results for each species, with a scale from 0 (disagreement) to 100 (agreement). Analyses were performed using R package FactoMineR (Husson et al., 2009).

### Identification of Ecogeographic Characteristics of CWR

In order to evaluate the pairwise degree of geographic overlap between sweetpotato CWR distribution models, we calculated an overlap measure equal to the frequency of shared 2.5 arc-minute geographic cells between taxa divided by the sum of the total number of cells in which either of the species are present (Kernohan et al., 2001; Fieberg and Kochanny, 2005). To assess the pairwise degree of ecogeographic niche overlap between species, we used Schoener’s index for niche similarity (D) and the adjusted similarity index (I) as outlined in Warren et al. (2008), using species distribution models and the species-specific subset of the 27 ecogeographic layers/ensemble models as inputs. Overlap indices range from 0 (no overlap) to 1 (complete overlap) and were implemented in the R package Phyloclim (Heibl, 2011).

We utilized ecogeographic information in combination with species presence data in order to identify populations of species with the potential for outstanding adaptations to climatic and/or edaphic conditions of interest to sweetpotato breeding objectives. We assessed the relative importance of the 27 ecogeographic variables (Supplementary Table S2) in explaining the total variation through a PCA after confirming the validity of its use through a Bartlett’s test performed in R package psych (Revelle, 2015). We created a hierarchical cluster of principal components (HCPC) in order to identify ecogeographic clusters for the sweetpotato wild relatives, driven by those variables demonstrating ≥15% difference (±) from the mean and a reduction of ≥20% from the mean standard deviation exhibited across all species, using R package FactoMineR. Boxplots for each of the 27 ecogeographic variables were created based upon CWR species occurrence data points, displaying the median and variance parameters per species per variable. Comparable ecogeographic variable data for the sweetpotato crop was extracted from area of cultivation maps (Monfreda et al., 2008) at a resolution of 5 arc-minutes, with a random sample of 1,000 points weighted by harvested area, taken from the major cultivation areas in Asia, Africa, and Latin America.

## Results

### Distributions of the Wild Relatives of Sweetpotato

**FIGURE 1:**
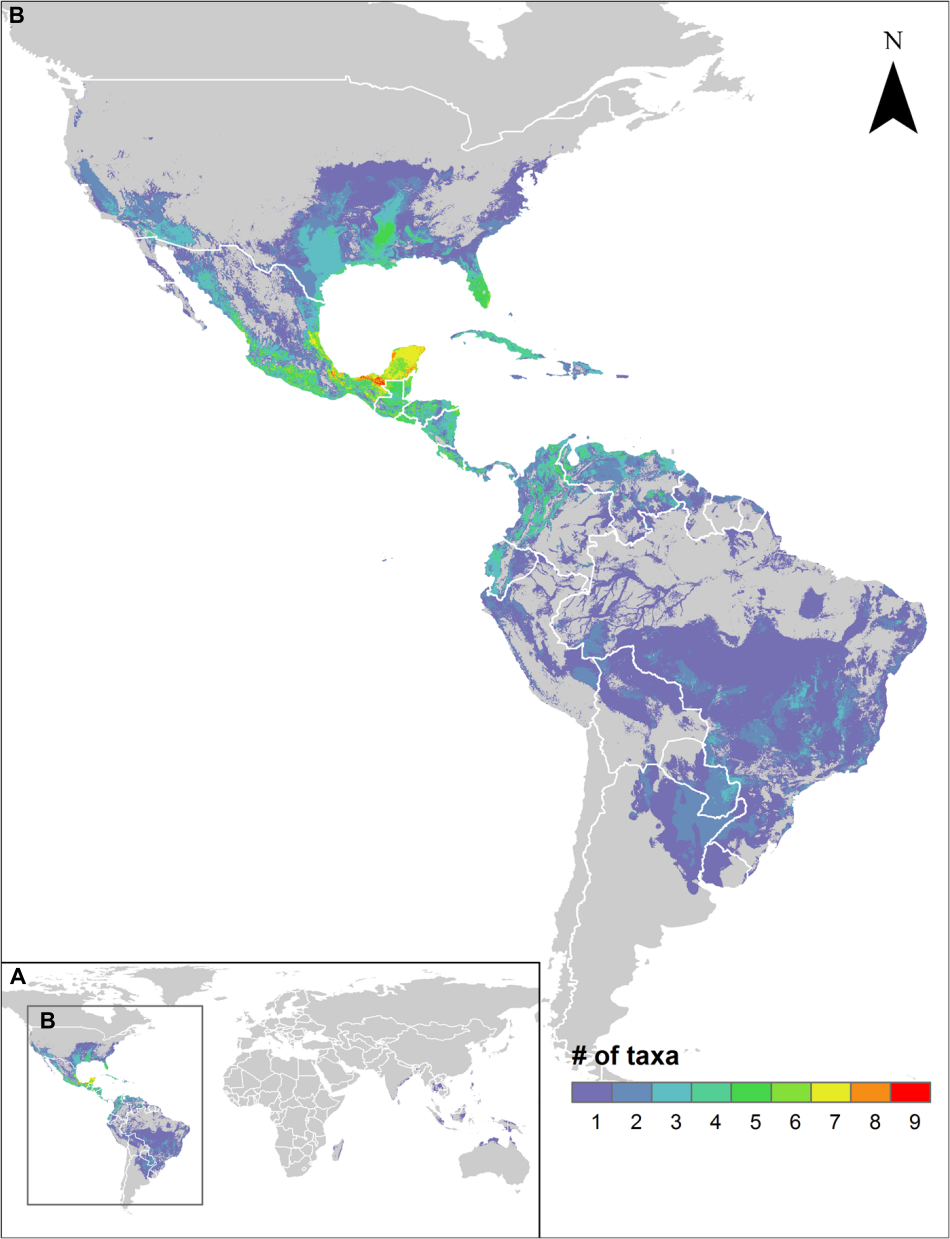
**Species richness map for assessed sweetpotato crop wild relative potential distribution models worldwide **(A)**, with concentration of species in the neotropics (B)**.

Sweetpotato CWR were modeled to occur from the central USA to northern Argentina in the Americas, including the Caribbean (Supplementary Figure S1). Species richness was greatest in central Mexico through Central America to the northern Andean region, and in the Southeastern USA, with up to nine species potentially overlapping in Mexico from the states of Veracruz through the Yucatan peninsula (**Figure 1**). The Mexican and Central American regions of distribution represent one of the posited centers of origin and primary diversity of cultivated sweetpotato (Austin, 1988; Austin and Huamán, 1996; Zhang et al., 2000; Gichuki et al., 2003; Roullier et al., 2013). Northwestern South America, with archeological remains of cultivated sweetpotato from Peru dating back to 8,000 years BP, which are among the oldest recorded domestication events on the continent (Piperno and Pearsall, 1998; Solis et al., 2001), displayed a considerably lesser but still notable degree of CWR species richness. One Old World species (*I. littoralis*; Austin, 1991) was also modeled to occur in coastal areas of Madagascar, South and Southeast Asia, Australia, and the Pacific region.

### Analysis of Current *Ex Situ* Conservation and Further Collecting Needs for CWR

**FIGURE 2:**
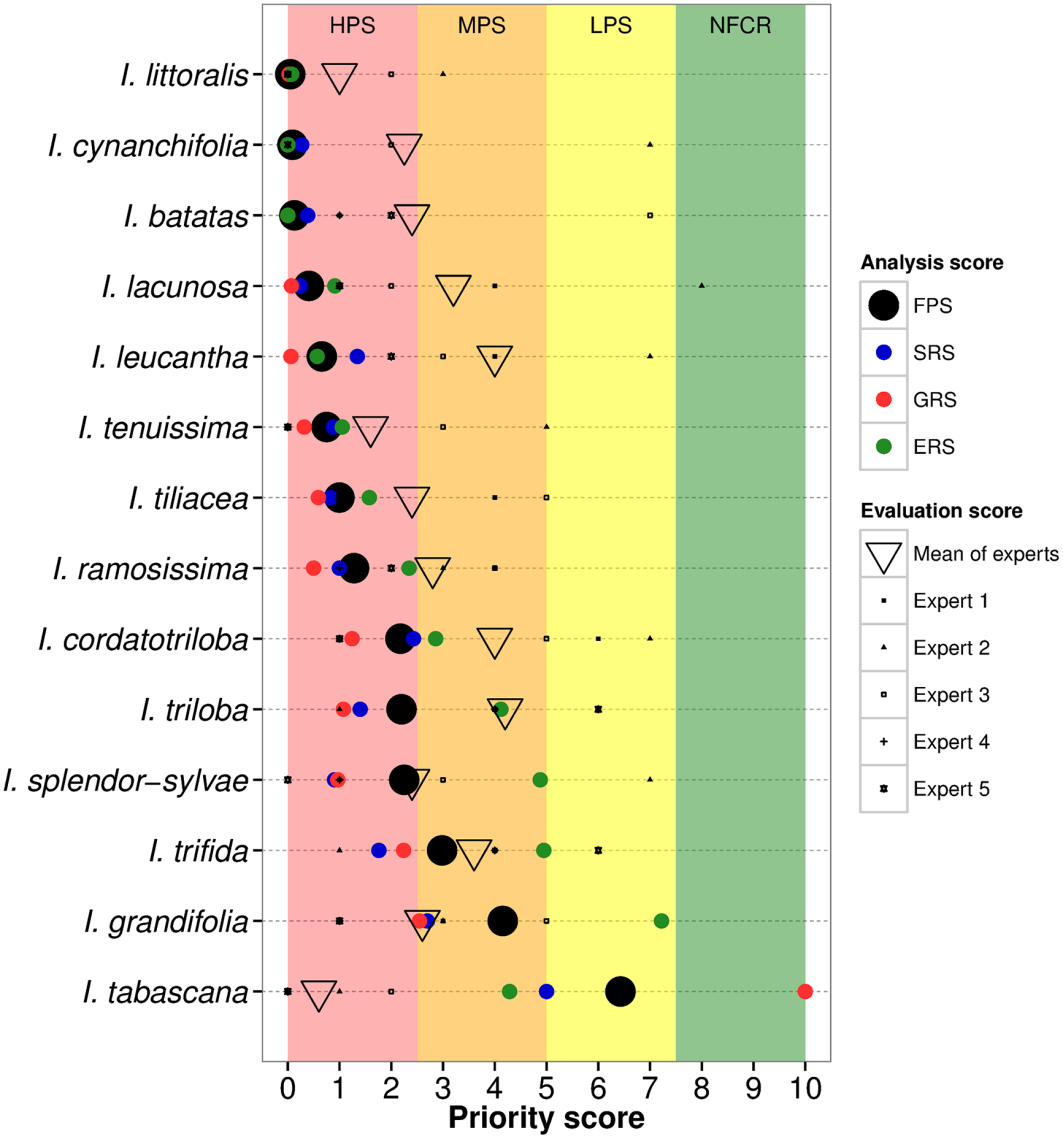
**Gap analysis results and comparable expert assessments per sweetpotato crop wild relative**. Species are listed by descending priority for further collecting by priority categories [high priority species, HPS (red); medium priority species, MPS (orange); low priority species, LPS (yellow); and no further collecting recommended, NFCR (green)]. The black circle represents the final priority score (FPS) for the species, which is the mean of the sampling representativeness score (SRS), geographic representativeness score (GRS), and ecological representativeness score (ERS).

Eleven out of the fourteen CWR species were assigned high priority for further collecting due to insufficient genebank accessions in comparison to the total number of occurrence samples (SRS), and to large geographic (GRS) and ecological (ERS) gaps in *ex situ* germplasm collections in comparison to the full potential distributions of the species (**Figure 2**, **Table 1**, Supplementary Figure S2). Six of these taxa are currently represented by ≤10 accessions conserved *ex situ*, moreover, with few exceptions these accessions lack associated geographic occurrence information (Supplementary Table S3). Total sampling representativeness and geographic coverage of species in germplasm collections were particularly lacking for taxa assessed high priority, while gaps in ecological representativeness were less extreme for some species (e.g., *I. cordatotriloba*, *I. triloba*, and *I. splendor-sylvae*). *Ipomoea grandifolia* and *I*. *trifida* were assessed as relatively well covered in regard to ecosystems represented *ex situ*, which raised their FPS into the medium priority for further collecting category. *Ipomoea tabascana* was assessed as of low priority for further collecting due to existing germplasm collections largely covering its very restricted distribution in southern Mexico, resulting in a high GRS score. The mean FPS across all CWR was 1.75 ± 1.82.

**Table 1:** Utilization characteristics, number of germplasm accessions conserved *ex situ*, further collecting priorities, and potential adaptive traits associated with ecogeographic niches of sweetpotato crop wild relatives.

Taxon	Gene pool	Ploidy	Germplasm accessions	Gap analysis priority	Mean expert priority	Eco geographic cluster	Potential adaptation to
*Ipomoea batatas*	2	4x = 60	4 (0)	HPS	HPS	2	Heat, high precipitation, drought, precipitation seasonality, clay soils
*I. cordatotriloba*	3	2x, 4x	103 (67)	HPS	MPS	1	Cold, temperature variation, clay soils, sandy soils
*I. cynanchifolia*	3	2x = 30	1 (0)	HPS	HPS	1,2	Drought, precipitation seasonality, sandy soils
*I. grandifolia*	3	2x = 30	124 (83)	MPS	MPS	1	Cold, temperature variation, clay soils, sandy soils
*I. lacunosa*	3	2x = 30	10 (1)	HPS	MPS	1	Cold, temperature variation, drought
*I. leucantha*	3	2x = 30	18 (15)	HPS	MPS	1,2	Heat, drought, precipitation seasonality, sandy soils
*I. littoralis*	2	2x = 30	2 (2)	HPS	HPS	2	Heat, high precipitation, drought, precipitation seasonality, sandy soils
*I. ramosissima*	3	2x = 30	34 (30)	HPS	MPS	2,1	Cold, high precipitation, clay soils
*I. splendor-sylvae*	3	2x = 30	16 (9)	HPS	HPS	2	Heat, high precipitation, drought, precipitation seasonality, clay soils
*I. tabascana*	2	4x = 60	4 (2)	LPS	HPS	2	Heat, high precipitation, clay soils
*I. tenuissima*	3	2x = 30	3 (1)	HPS	HPS	1	Heat, cold, temperature variation, sandy soils
*I. tiliacea*	3	4x = 60	61 (44)	HPS	HPS	2	Heat, high precipitation, clay soils
*I. trifida*	2	2x,3x,4x,6x	248 (159)	MPS	MPS	2	Heat, high precipitation, drought, precipitation seasonality
*I. triloba*	3	2x = 30	121 (74)	HPS	MPS	2,1	Heat, drought

*Ploidy data adapted from Nimmakayala et al. (2011). Germplasm accessions displays both the total number of accessions recorded in genebanks, as well as the number of accessions with unique geographic coordinates (i.e., unique populations) in parenthesis. HPS = high, MPS = medium, and LPS = low priority species for further collecting.*

**FIGURE 3:**
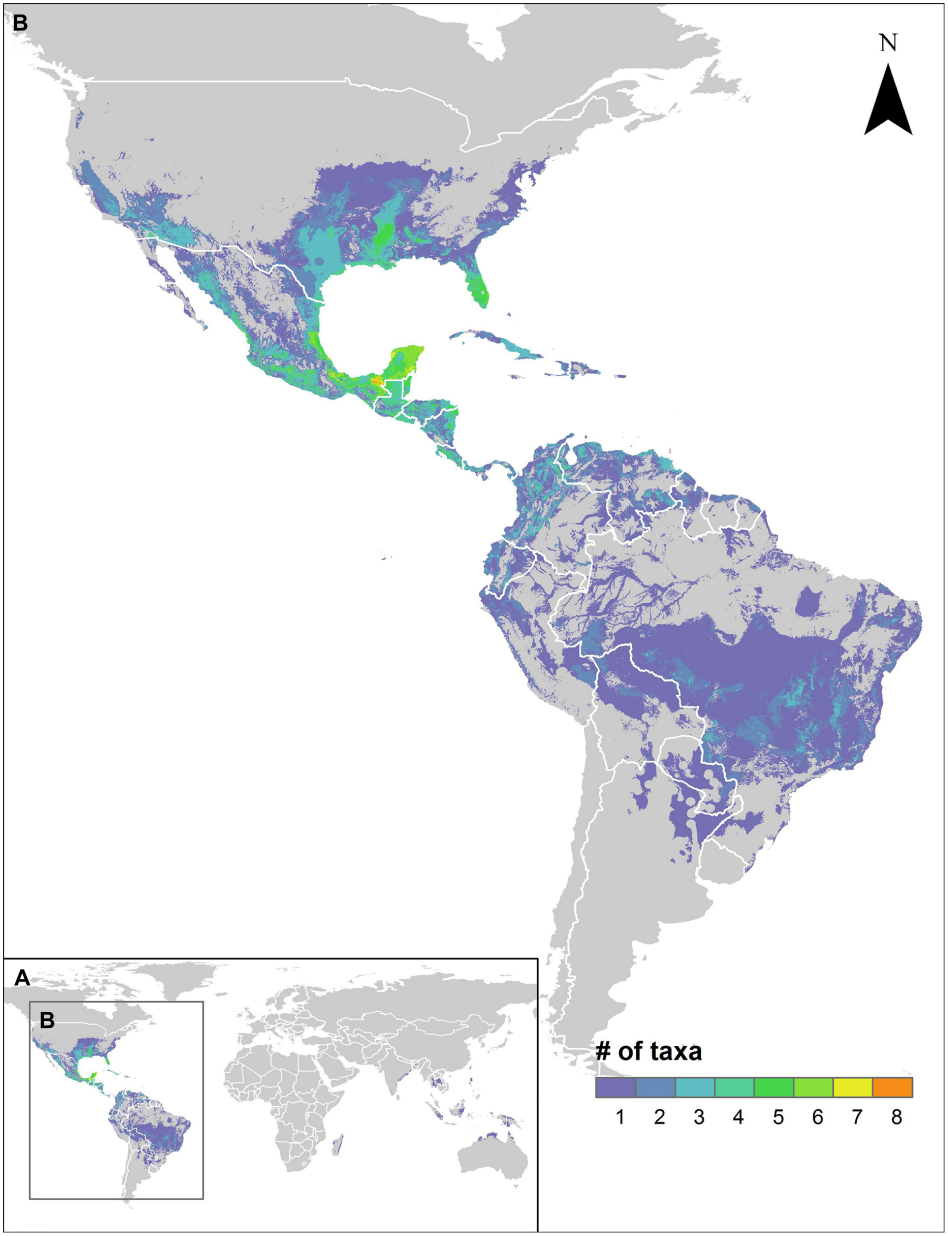
**Further collecting priorities hotspots map for high priority species (HPS) sweetpotato crop wild relatives. The map displays areas worldwide (A)** within the potential distributions of HPS species that have not been previously collected for *ex situ* conservation, with concentration of species in the neotropics **(B)**.

Paralleling the distribution of species richness of sweetpotato CWR, the regions identified for further collecting of the greatest number of species included central and southern Mexico and the Southeastern USA, with up to seven species prioritized for further collecting occurring in the same area (**Figure 3**). Further collecting priorities were recognized in a total of 50 countries throughout the range of the genepool (Supplementary Figure S3, Supplementary Table S4). Occurrence data, potential distribution models, and collecting priorities maps for all modeled species are available in an interactive map format at http://www.cwrdiversity.org/distribution-map/.

### Expert Evaluation of Conservation Assessment Results

The average of the directly comparable EPS across experts was 2.65 (±1.10) as a mean across species, varying 1.95 points on average from the FPS, with seven taxa designated by the experts as HPS, and seven as MPS (**Table 1**, **Figure 2**). For most species, this mean was highly influenced by one or two experts’ giving species considerably less priority than the other experts. Species with closest accord between the gap analysis results and the comparable expert analysis included *I. splendor-sylvae*, *I. trifida*, *I. tenuissima*, *I. littoralis*, *I. tileacea*, *I. ramosissima*, and *I. grandifolia*. Taxa with the greatest difference between gap analysis and comparable expert values included *I. tabascana* and *I. leucantha*.

Two species were assessed by the experts as higher priority for further collecting than the results of the gap analysis. Although *ex situ* collections for the highly restricted distribution of *I. tabascana* were determined in the gap analysis to be fairly comprehensive (LPS), the experts assigned the species HPS for further collecting due to its very limited overall number of germplasm holdings, and to the difficulty in producing viable seed in *ex situ* conservation. *Ipomoea grandifolia* was assessed in the gap analysis as reasonably comprehensively represented in regard to ecosystem diversity, and thus assigned medium priority for further collecting, while the experts gave moderately higher priority to the species.

The contextual expert priority score per species, which also included the expert’s knowledge of threats *in situ* as well as usefulness for crop improvement, did not vary widely from the comparable score [mean across all experts and species was 2.76 (±0.85); mean difference between comparable and contextual expert scores across all species and experts was 0.11]. Due largely to differences between the perceptions of relative sufficiency in regard to the overall number of germplasm accessions by the experts versus gap analysis results (e.g., for *I. tabascana*, *I. triloba*, and *I. leucantha*), the comparable and contextual assessments did not correlate well with the gap analysis results for the genepool as a whole (Supplementary Figures S4A,B).

The multiple factor analysis combining the comparable expert priority score, contextual expert priority score, evaluation of gap analysis results score, evaluation of occurrence data, evaluation of potential species distribution models, and evaluation of collecting priorities map, revealed sufficient agreement among experts and variables to justify the use of the expert evaluation index, although variation in expert option was notable for many species. Data inputs and resulting distribution models were generally assessed positively as a mean across experts, with eight species receiving very positive index values. Those species with the highest accord between all variables and experts and the gap analysis results included *I. littoralis*, *I. spendor-sylvae*, *I. ramosissima*, *I. tenuissima*, and *I. tiliacea*. Those species with the lowest accord included *I. tabascana*, *I. triloba*, and *I. grandifolia* (Supplementary Figure S4). FPS results were particularly influenced by spotty occurrence records for the majority of species, with gaps recognized by the experts.

### Identification of Ecogeographic Characteristics of CWR

The analysis of geographic and ecogeographic accord between pairwise potential species distribution models segregated species well into temperate North American (e.g., *I. lacunosa*, *I. tenuissima*), Mesoamerican (e.g., *I. splendor-sylvae*, *I. tabascana*), widely distributed tropical (e.g., *I. triloba*, *I. trifida*), and South American (e.g., *I. grandifolia*, *I. cynanchifolia*) areas (Supplementary Figure S5).

Strong linear relationships were found between bioclimatic variables within the study area, justifying the application of the PCA, with 75.6% of variance explained through four principal components (Supplementary Figure S6). The first principal component (32% of variation) was correlated with temperature extremes and fluctuation. The second component (21.6% of variation) was most occupied by precipitation variables related to drought. The third component (13.9% of variation), was related with altitude, and the final component (8.1%) with soil texture characteristics.

Species occurrence data segregated into temperate and tropical ecogeographic clusters, with the great majority of species’ distributions strongly occurring within a single cluster. Ecogeographic variables most strongly influencing the definition of the temperate cluster (1) included those associated with temperature variation and relatively low precipitation, elevation, and soil organic matter. The most determinant variables in the tropical cluster (2) were related to relatively high and consistent temperatures. Those species displaying a notable proportion of occurrences within both clusters included *I. cynanchifolia* and *I. triloba*, and to a lesser degree *I. leucantha* and *I. ramosissima* (**Table 1**, Supplementary Figure S6).

**FIGURE 4:**
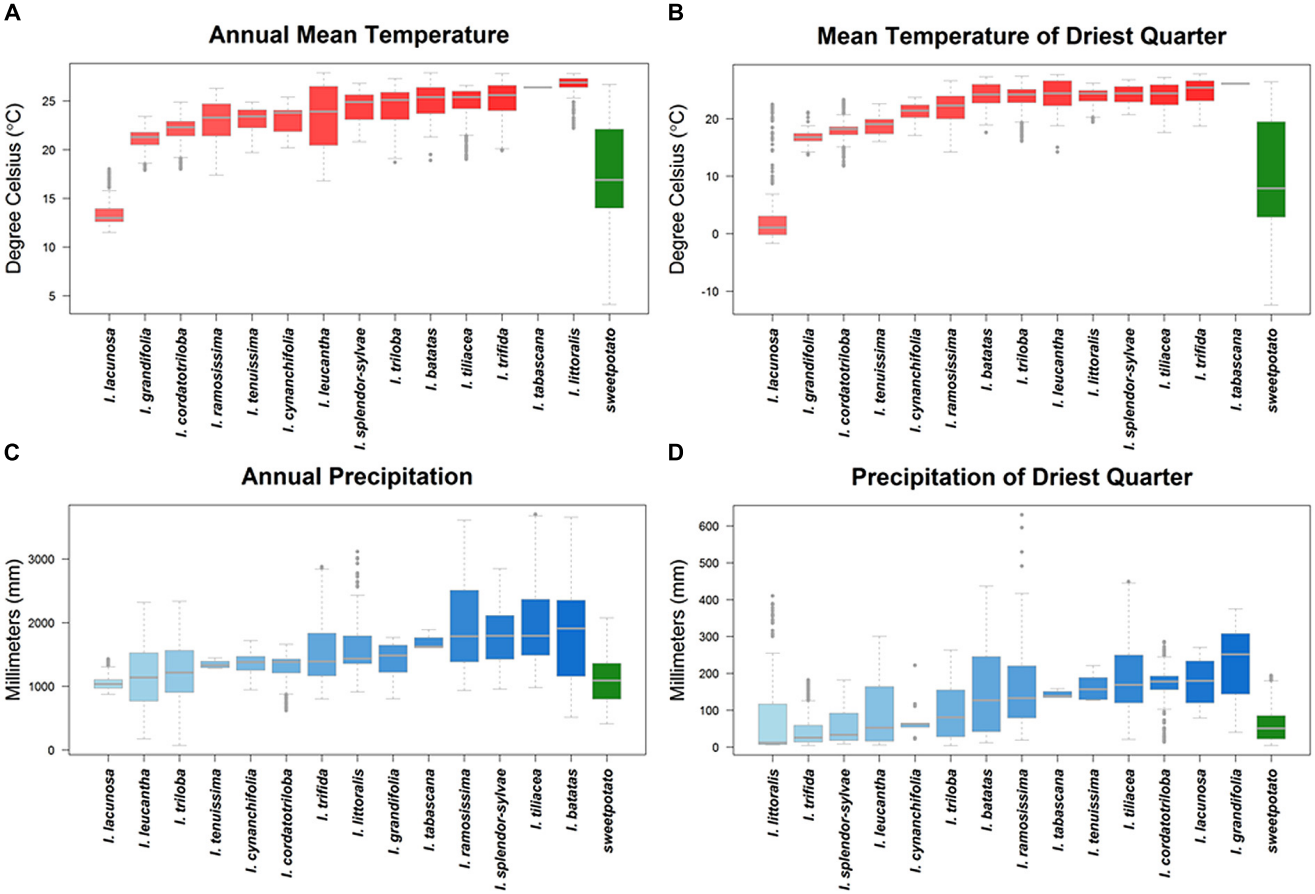
**Ecogeographic niches of crop wild relative species based upon their occurrence data presence locations, and the sweetpotato crop, for **(A)** annual mean temperature, **(B)** mean temperature of the driest quarter of the year, **(C)** annual precipitation, and **(D)** precipitation of the driest quarter of the year**. The thick gray line represents median values, boxplots between 25 and 75% variation, and open circles outliers within 90% of total variation. For a principal component analysis of variables see Supplementary Figure S6 and for ecogeographic niches displaying total variation for all variables per species see Supplementary Figure S7.

Ecogeographic niche assessments of sweetpotato CWR based upon occurrence data points revealed large differences in potential adaptation to temperature, precipitation, and edaphic characteristics (**Table 1**, **Figure 4**, Supplementary Figure S7). Such adaptation for many species fell well outside the modeled niche of the cultivated species, particularly for high temperatures both in wet and dry conditions, as well as high precipitation. Species of notable adaptation to high mean annual, monthly, and quarterly temperatures included *I. littoralis*, *I. tabascana*, *I. trifida*, *I. leucantha*, *I. tiliacea*, *I. tenuissima*, *I. triloba*, *I. splendor-sylvae*, and wild *I. batatas*. USA species *I. lacunosa* stood out for adaptation to low temperatures, with *I. grandifolia*, *I. cordatotriloba*, *I. tenuissima*, and *I. ramossissima* also demonstrating cold tolerance. These same species were among those displaying the widest adaptation to temperature fluctuation.

Crop wild relative of sweetpotato occurring in areas of high precipitation included *I. ramossissima*, *I. littoralis*, *I. splendor-sylvae*, *I. tabascana*, *I. tiliacea*, *I. trifida*, and wild *I. batatas*. *Ipomoea littoralis*, *I. trifida*, *I. splendor-sylvae*, *I. leucantha*, *I. cynanchifolia*, *I. triloba*, *I. lacunosa*, and wild *I. batatas* were distributed in regions with low precipitation. These species were also among those displaying the widest adaptation to precipitation seasonality. Sweetpotato CWR also displayed variation in adaptation to edaphic characteristics. *Ipomoea tabascana*, *I. grandifolia*, *I. tiliacea*, *I. splendor-sylvae*, *I. ramosissima*, *I. cordatotriloba*, and wild *I. batatas* occurred in clay soils, while *I. tenuissima*, *I. littoralis*, *I. cynanchifolia*, *I. grandifolia*, *I. leucantha*, and *I. cordatotriloba* were distributed in sandy soils.

## Discussion

This article utilizes the most current knowledge on species concepts within *I*. series *Batatas*. Due to taxonomic uncertainties and to the notable dearth of study material for sweetpotato CWR, the results represent a preliminary understanding of the geography and conservation status of the series, to be further refined following increased levels of collecting sufficient to support the needed taxonomic and crossability research. Further collecting of germplasm thus serves two purposes: (a) conserves genetic resources for the long-term and makes these resources available to breeders; and (b) provides the basic materials needed by researchers to understand the diversity present in the CWR of sweetpotato.

A total of 78.6% of the wild relatives of sweetpotato considered in this study were assessed as high priority for further collecting for *ex situ* conservation. With general agreement from expert evaluators of medium to high importance for all species, it is clear that much remains to be done to safeguard the wild genetic resources of this critically important root crop. Included in this list of priorities are species with very few germplasm accessions accessible to the global community in genebank information systems, including *I*. *cynanchifolia, I*. *littoralis, I*. *tenuissima, I*. *tabascana, I*. *lacunosa, I*. *leucantha, I*. *splendor-sylvae*, and clearly designated wild forms of the crop conspecific *I. batatas*. Such species represent the highest level of priority for further collecting for use in systematic analyses as well as genetic resources conservation. As the species diversity gaps in genebank collections largely align with the geographic distribution of species richness of sweetpotato CWR, hotspots in central Mexico to Central America, and in the extreme Southeastern USA, represent particularly high priority regions for efficient collecting of the sweetpotato genepool (**Figure 3**). Additional unrepresented populations of HPS such as *I. littoralis* and *I. cynanchifolia* occur outside those regions, thus targeted collecting throughout the geographic distribution of the genepool is necessary in order to form comprehensive germplasm collections.

Due to having relatively large potential distributions occupying a diversity of ecosystems, species such as *I*. *triloba*, *I. cordatotriloba*, and *I*. *tiliacea* were categorized as high priority, and *I*. *trifida* and *I*. *grandifolia* as medium priority for further collecting despite having sizable currently existing germplasm collections. As the cost of conserving and investigating germplasm *ex situ* is significant, a further assessment of what constitutes sufficient representation of these species in germplasm collections is warranted. Given adequate resources, further collecting may be worthwhile, as extremely valuable traits sourced from CWR of some crops have been found in only a few populations despite screening of a large number of accessions (Brar and Khush, 1997), and accessions of individual CWR species such as *I*. *triloba* have been shown to possess notable variation in traits such as tolerance to precipitation (Martin and Jones, 1973; Nimmakayala et al., 2011).

As Maxent models are based upon known presence points for species and are thus subject to sampling bias, they may not fully capture the possibility of occurrence of populations of CWR species in unique climates (Araújo and Guisan, 2006; Loiselle et al., 2007; Kramer-Schadt et al., 2013). Further field exploration of climatic extremes beyond the edges of the distributions created through these methods may therefore lead to the discovery of new populations with particularly valuable adaptations to abiotic stress (Williams et al., 2009). Investigation of non-native populations (e.g., *I. trifida* in Asia) may also yield novel adaptations of use in crop improvement. As techniques for the utilization of distantly related germplasm improve, the exploration of other *Ipomoea* species outside of *I*. series *Batatas* may also result in the identification of beneficial traits [e.g., *Ipomoea purpurea* (L.) Roth, for stem nematode and SPVD resistance (Cao et al., 2009)].

Analysis of geographic overlap and ecogeographic similarities between species, as well as ecogeographic clusters among all species, can supplement morphological and genetic analyses in differentiating useful genetic resources, and can serve as a point of departure for identifying taxonomically problematic populations for further investigation. These analyses may also indicate geographic areas of particular interest in regard to high rates of hybridization, as in the case of *I*. *cordatotriloba* and *I*. *lacunosa* (Duncan and Rausher, 2013), which indeed were identified as sharing similar geographies and ecogeographic niches. The purported hybrid descendent of these species, *I. leucantha*, was modeled as containing a much more extended latitudinal gradient in the northern hemisphere than its parents, as well as a differing ecogeographic niche, including potential adaptation to high heat and to drought.

Genetic resistance is essential to efforts to overcome major biotic and abiotic constraints in sweetpotato production. As these constraints are often interrelated, e.g., drought stress with SPW and SPVD damage, enhancement of broad resistance for traits such as drought may improve yield across relatively large geographic areas, without the need to breed for resistance to localized viral strains (Ngailo et al., 2013). Such broad tolerance may also improve adoption rates for sweetpotato varieties with other desirable characteristics, such as high β-carotene content.

Reliable funding for germplasm collections is paramount in order to safeguard sweetpotato CWR genetic resources in the long-term and to continue to make *ex situ* collections available to the global community. Further investment in genebank information systems, *ex situ* conservation technologies (i.e., storage, testing, and regeneration), safety duplication of unique germplasm, and ensuring facilitated access to genetic diversity is equally essential (Food and Agriculture Organization of the United Nations [FAO], 2002, 2010; Khoury et al., 2010). In order to maximize the usefulness of conserved germplasm, characterization and evaluation for traits of interest, and increased breeding research, which have been limited for CWR of sweetpotato, are also needed. Further research combining morphological studies, trait evaluations, and genetic diversity analyses is likewise critically needed for elucidating species boundaries and highlighting accessions of particular value for use in breeding. Recent focused research has produced quick gains, including the identification of new species (Duncan and Rausher, 2013; Wood et al., in press). Through these actions the crop research community will contribute to ensuring the long term viability of this important root crop.

## Author Contributions

CK, BH, NC-A, HA, CS, HJ, and SdH conceived and designed the study. CK, NC-A, HA, CS, and VB gathered data and performed the analyses. CK, BH, NC-A, HA, CS, RM, RS, JW, GR, LE, RJ, CY, SdH, and PS analyzed the results. CK and BH wrote the manuscript. CK, BH, NC-A, HA, CS, RM, RS, JW, GR, LE, RJ, CY, VB, HJ, SdH, and PS edited the manuscript.

## Conflict of Interest Statement

The authors declare that the research was conducted in the absence of any commercial or financial relationships that could be construed as a potential conflict of interest.
