# Supplementary material for: Distributions, ex situ conservation priorities, and genetic resource potential of crop wild relatives of sweetpotato [Ipomoea batatas (L.) Lam., I. series Batatas]
Source: Front Plant Sci. 2015 Apr 21;6:251. doi: 10.3389/fpls.2015.00251 (PMC4404978; doi:10.3389/fpls.2015.00251)
Supplement: Supplementary file 1 [file Data_Sheet_1.DOCX]

**Supplementary Material**

Article title: Distributions, *ex situ* conservation priorities, and genetic resource potential of crop wild relatives of sweetpotato [*Ipomoea batatas* (L.) Lam., *I*. series *Batatas*]

Authors: Colin K. Khoury, Bettina Heider, Nora P. Castañeda-Álvarez, Harold A. Achicanoy, Chrystian C. Sosa, Richard E. Miller, Robert W. Scotland, John R.I. Wood, Genoveva Rossel, Lauren A. Eserman, Robert L. Jarret, G. Craig Yencho, Vivian Bernau, Henry Juarez, Steven Sotelo, Stef de Haan, and Paul C. Struik

The following Supplementary Material is available for this article:

**Figure S1** Sweetpotato [*Ipomoea batatas* (L.) Lam.] crop wild relative potential distribution models

**Figure S2** Distribution of priority scores per sweetpotato [*Ipomoea batatas* (L.) Lam.] crop wild relative species for (a) sampling representativeness score (SRS), (b) geographic representativeness score (GRS), and (c) ecological representativeness score (ERS).

**Figure S3** Number of sweetpotato [*Ipomoea batatas* (L.) Lam.] crop wild relatives prioritized for further collecting for *ex situ* conservation per country. HPS = high, MPS = medium, and LPS = low priority species for further collecting.

**Figure S4** Expert evaluation accordance with gap analysis results: (a) correlation between gap analysis results and comparable expert evaluation scores, (b) correlation between gap analysis results and contextual expert evaluation scores, (c) correlation circle of all evaluation variables, (d) combined expert evaluation index score per sweetpotato [*Ipomoea batatas* (L.) Lam.] crop wild relative.

**Figure S5** Geographic overlap and ecogeographic similarity of potential distribution models between sweetpotato [*Ipomoea batatas* (L.) Lam.] crop wild relative species: (a) geographic overlap of potential distribution models, (b) ecogeographic similarity index (I) of potential distribution models.

**Figure S6** Principal component analysis of ecogeographic variables associated with occurrence data for sweetpotato [*Ipomoea batatas* (L.) Lam.] crop wild relatives: (a) variation represented in the first four principal components, (b) determining bioclimatic variables for the first four principle components, (c) contribution of cluster identified occurrence data points per species, (d) geographic origin of clustered occurrence data points.

**Figure S7** Ecogeographic niches of crop wild relative (CWR) species based upon their occurrence data presence locations, and the sweetpotato [*Ipomoea batatas* (L.) Lam.] crop, per bioclimatic and edaphic variable.

**Table S1** Sources of occurrence data for assessed sweetpotato [*Ipomoea batatas* (L.) Lam.] crop wild relative species.

**Table S2** Ecogeographic variables utilized in sweetpotato [*Ipomoea batatas* (L.) Lam.] crop wild relative potential distribution modeling and climatic niche analyses.

**Table S3** Gap analysis and comparable expert evaluation priorities results per sweetpotato [*Ipomoea batatas* (L.) Lam.] crop wild relative.

**Table S4** Countries identified for further collecting per sweetpotato [*Ipomoea batatas* (L.) Lam.] crop wild relative.

*Figures*


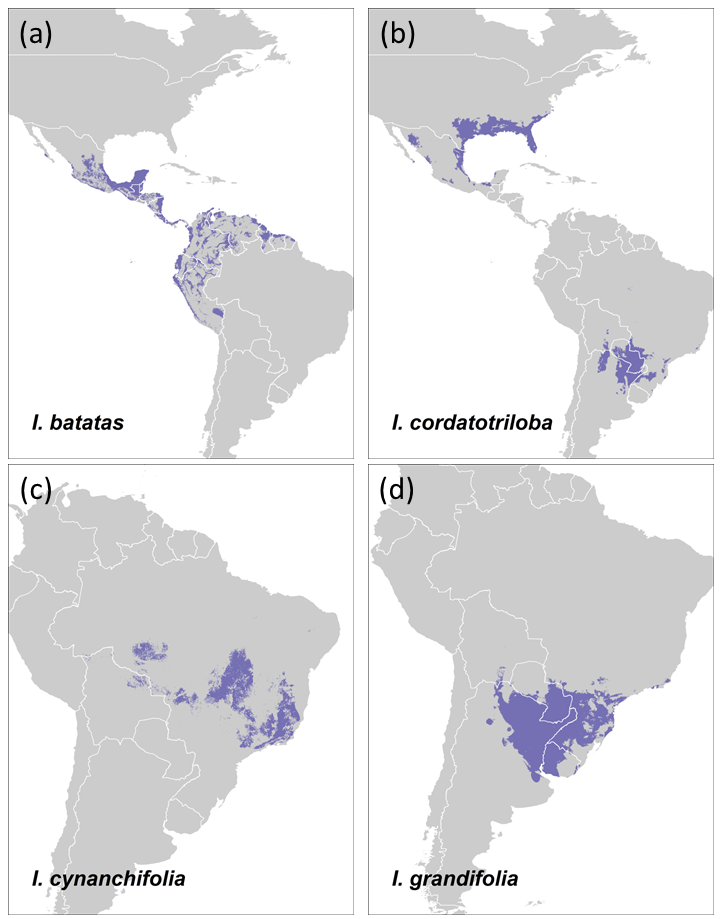


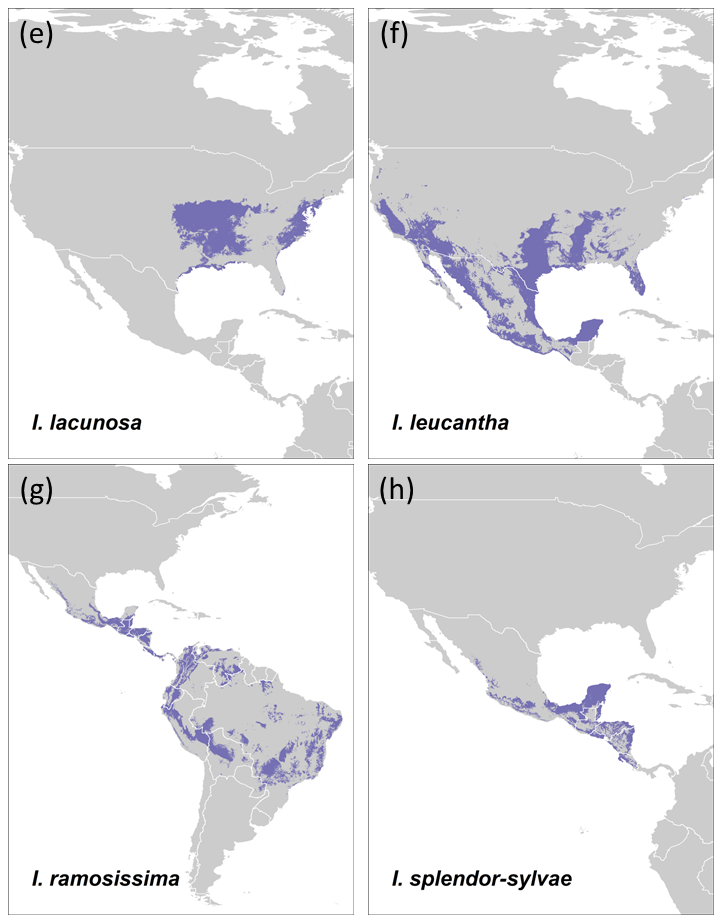


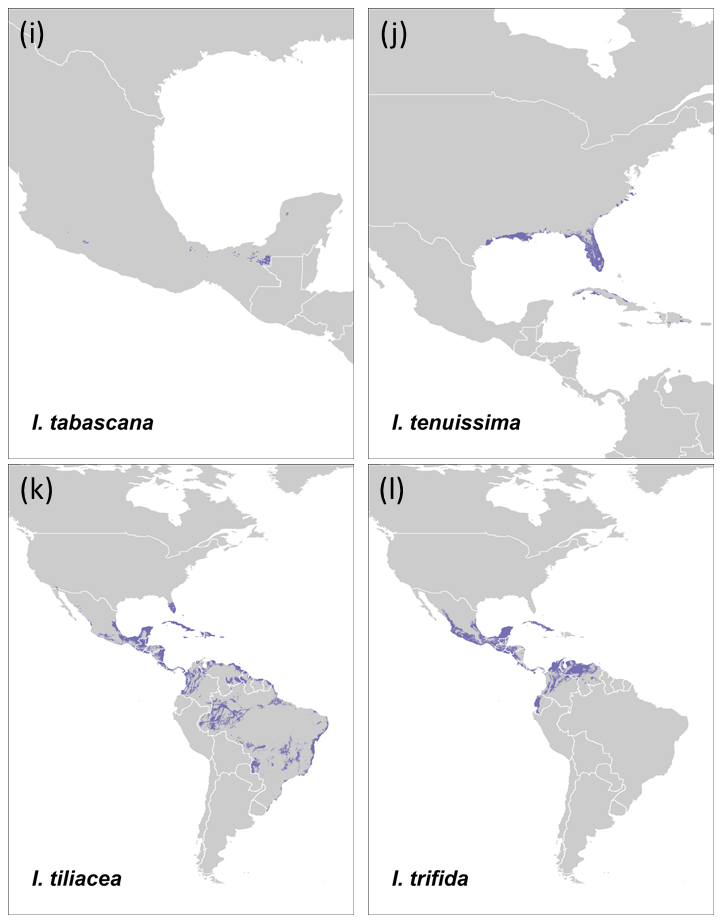


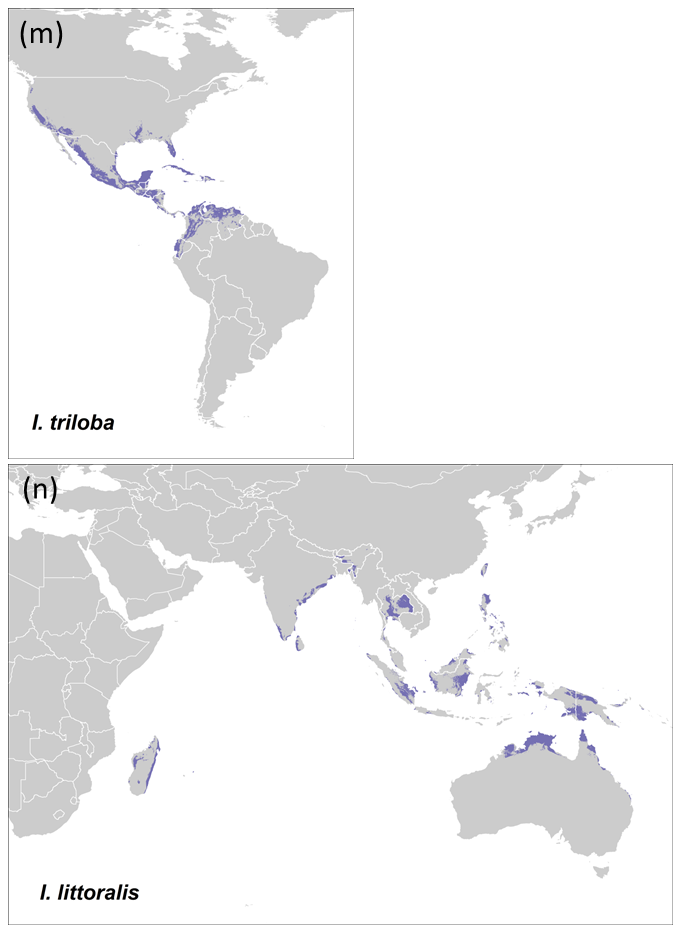


**Figure S1** Sweetpotato [*Ipomoea batatas* (L.) Lam.] crop wild relative potential distribution models


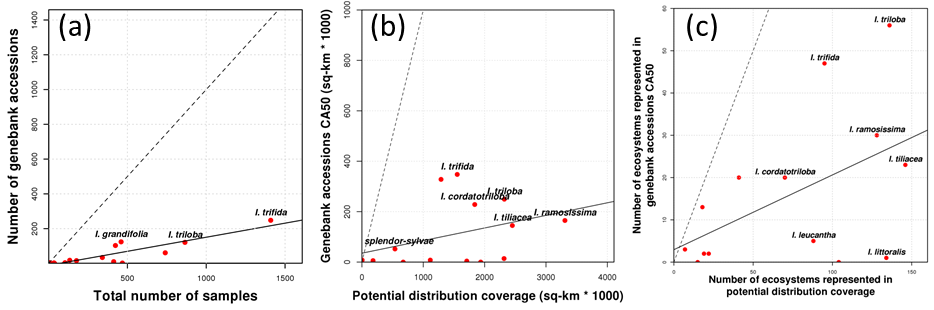


**Figure S2** Distribution of priority scores per sweetpotato [*Ipomoea batatas* (L.) Lam.] crop wild relative species for (a) sampling representativeness score (SRS), (b) geographic representativeness score (GRS), and (c) ecological representativeness score (ERS).


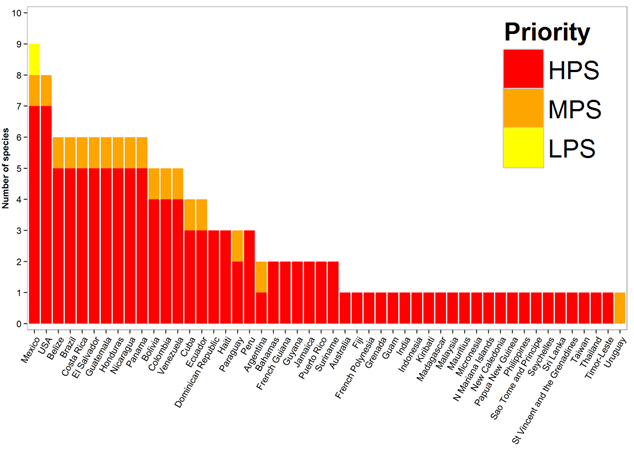


**Figure S3** Number of sweetpotato [*Ipomoea batatas* (L.) Lam.] crop wild relatives prioritized for further collecting for *ex situ* conservation per country. HPS = high, MPS = medium, and LPS = low priority species for further collecting.


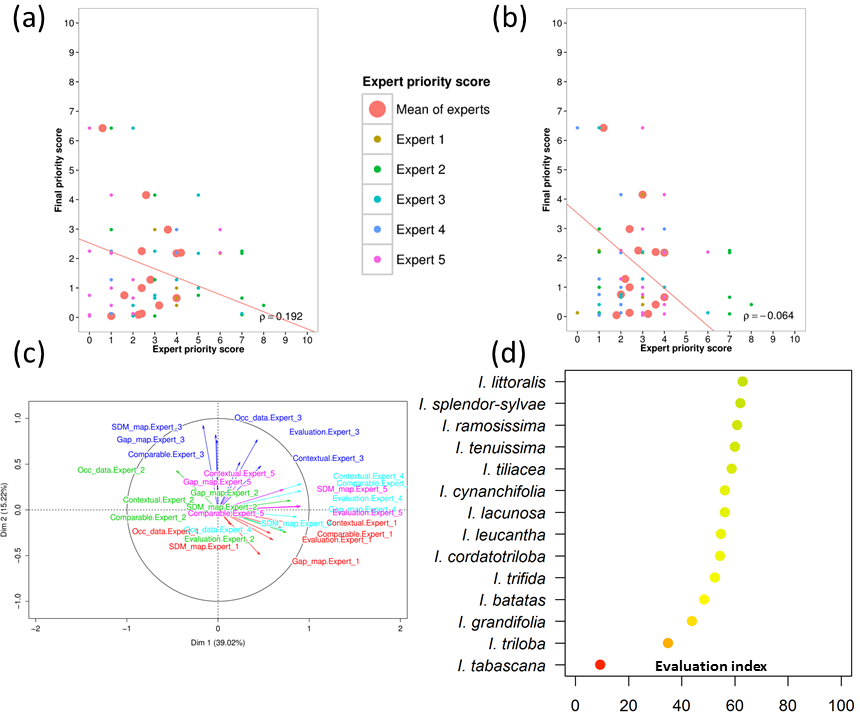


**Figure S4** Expert evaluation accordance with gap analysis results: (a) correlation between gap analysis results and comparable expert evaluation scores, (b) correlation between gap analysis results and contextual expert evaluation scores, (c) correlation circle of all evaluation variables [comparable expert priority score (Comparable), contextual expert priority score (Contextual), evaluation of gap analysis results score (Evaluation), evaluation of occurrence data (Occ_data), evaluation of potential species distribution models (SDM_map), and evaluation of collecting priorities map (Gap_map)], (d) combined expert evaluation index score per sweetpotato [*Ipomoea batatas* (L.) Lam.] crop wild relative.


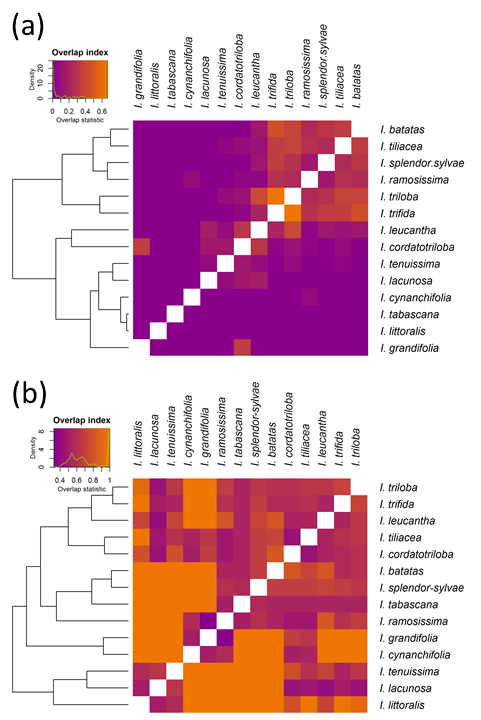


**Figure S5** Geographic overlap and ecogeographic similarity of potential distribution models between sweetpotato [*Ipomoea batatas* (L.) Lam.] crop wild relative species: (a) geographic overlap of potential distribution models, (b) ecogeographic similarity index (I) of potential distribution models.


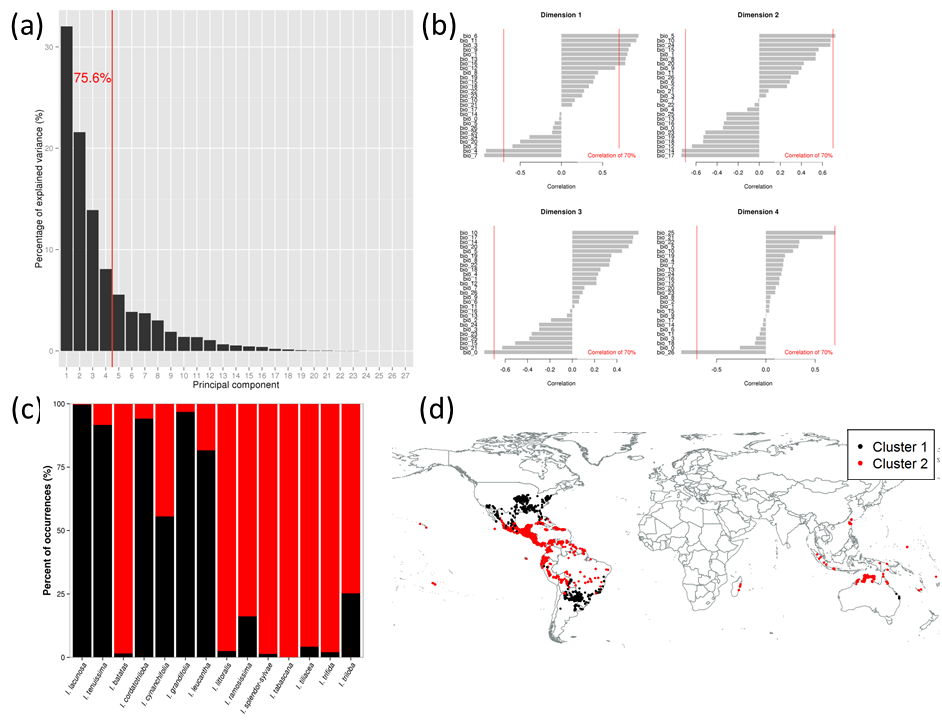


**Figure S6** Principal component analysis of bioclimatic variables associated with occurrence data for sweetpotato [*Ipomoea batatas* (L.) Lam.] crop wild relatives: (a) variation represented in the first four principal components, (b) determining bioclimatic variables for the first four principle components, (c) contribution of cluster identified occurrence data points per species, (d) geographic origin of clustered occurrence data points.


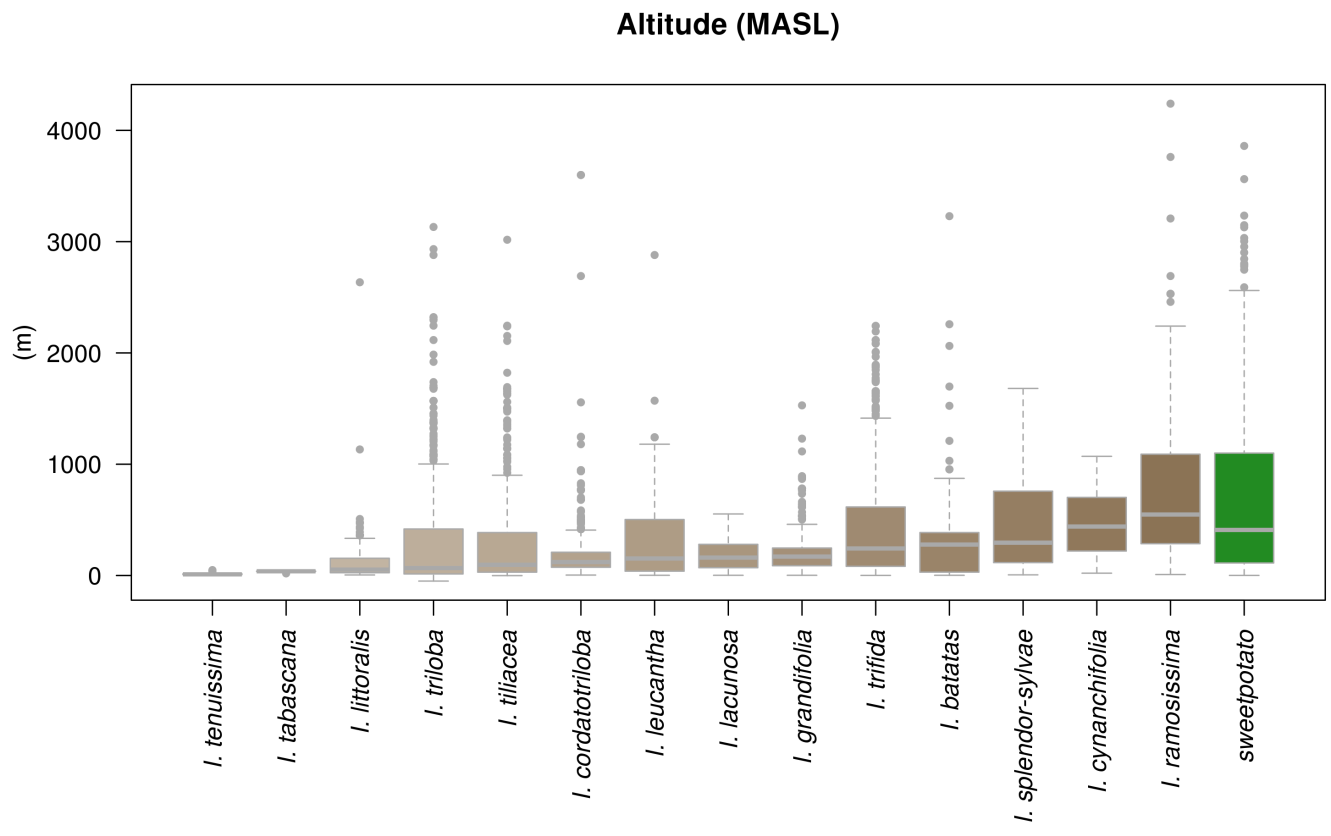


**Figure S7** Ecogeographic niches of crop wild relative (CWR) species based upon their occurrence data presence locations, and the sweetpotato [*Ipomoea batatas* (L.) Lam.] crop, per bioclimatic and edaphic variable. The bold grey line represents median values, boxplots between 25-75% variation, and circles outliers. Fig. S7A Niches of CWR species and the sweetpotato crop for altitude.


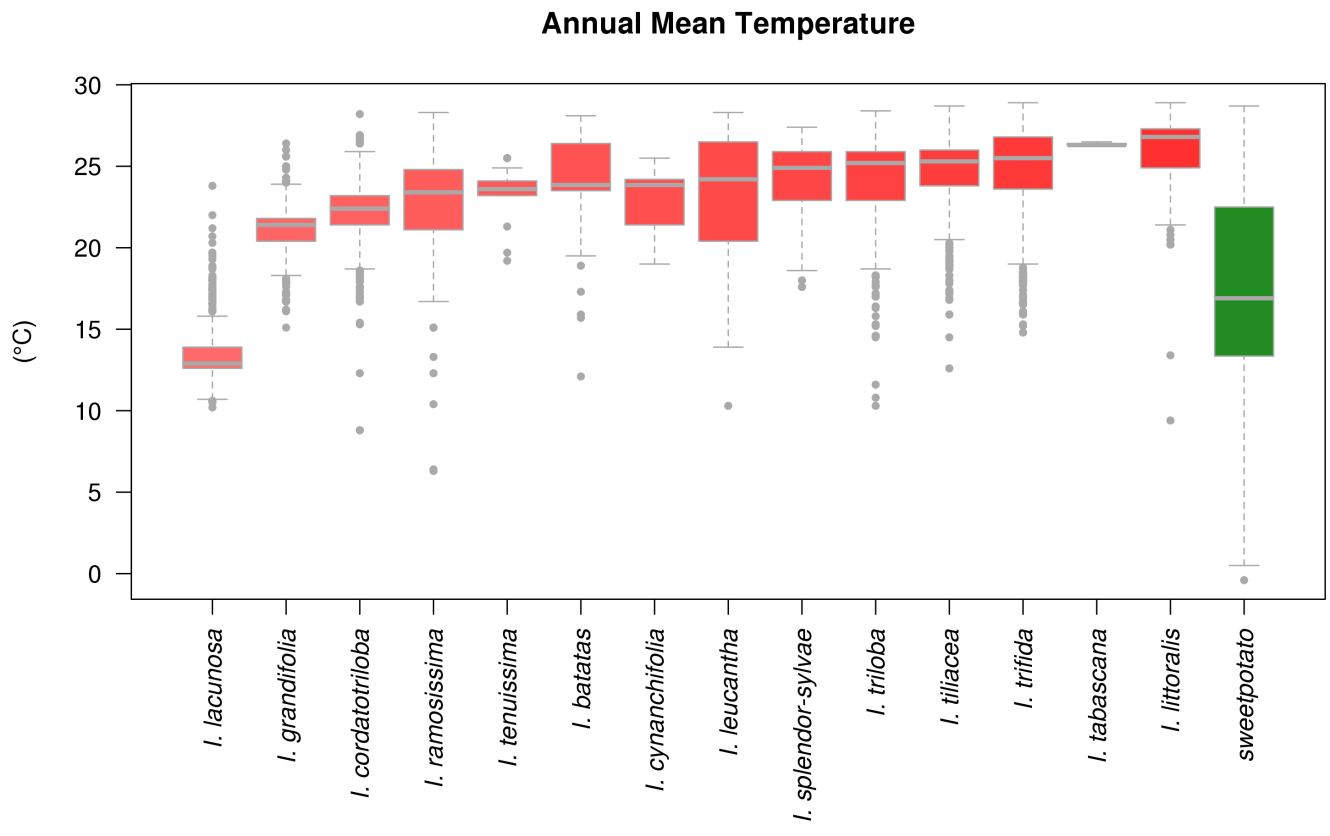


Figure S7B Climatic niches of CWR species and the sweetpotato crop for annual mean temperature.


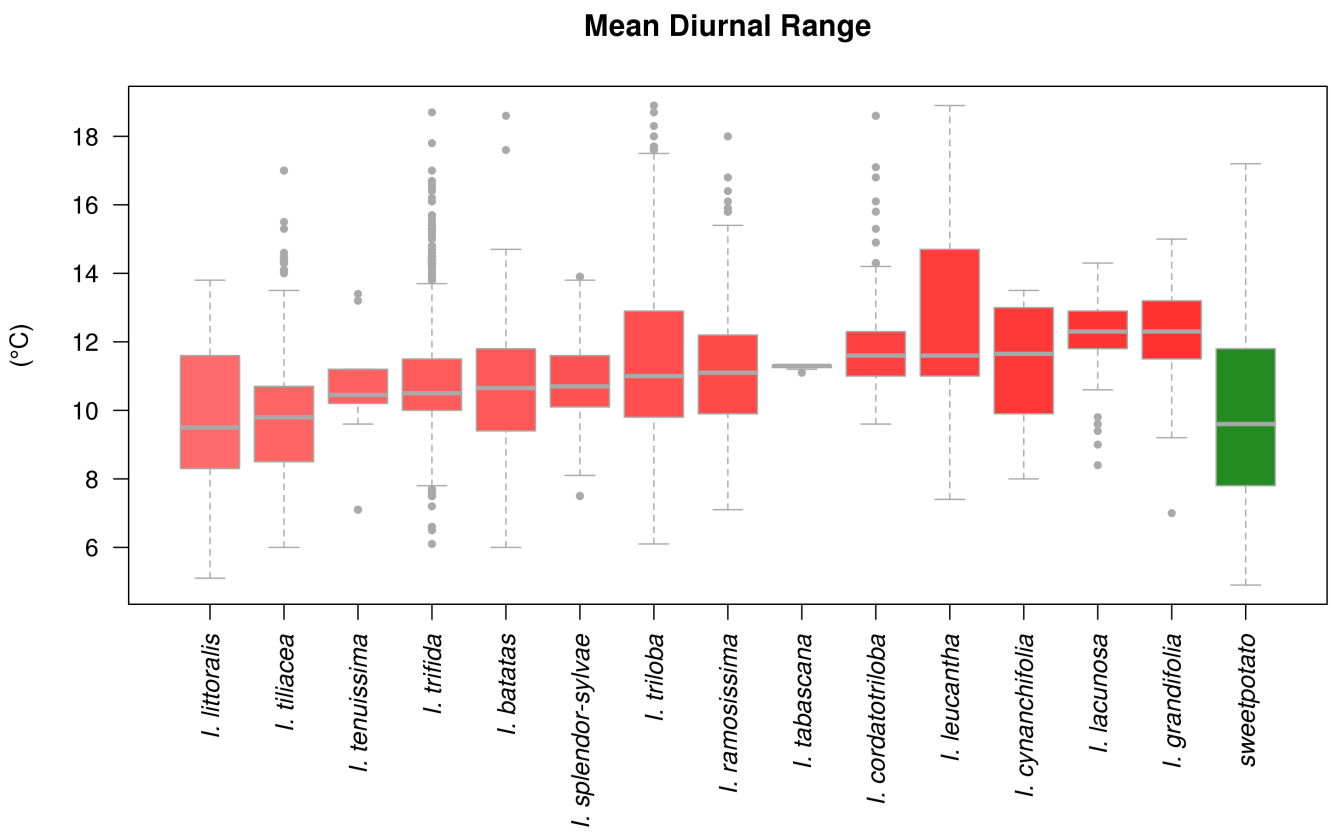


Figure S7C Climatic niches of CWR species and the sweetpotato crop for mean diurnal range.


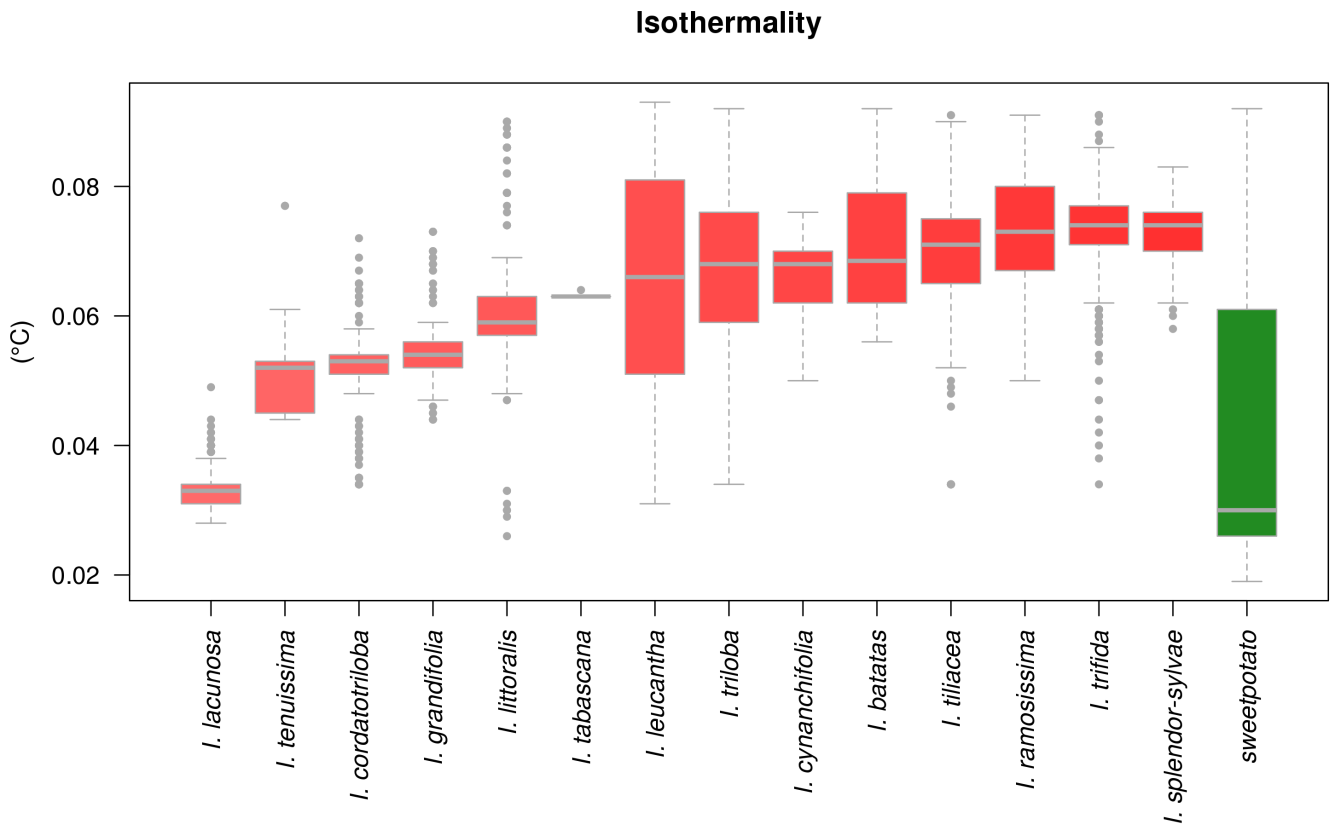


Figure S7D Climatic niches of CWR species and the sweetpotato crop for isothermality.


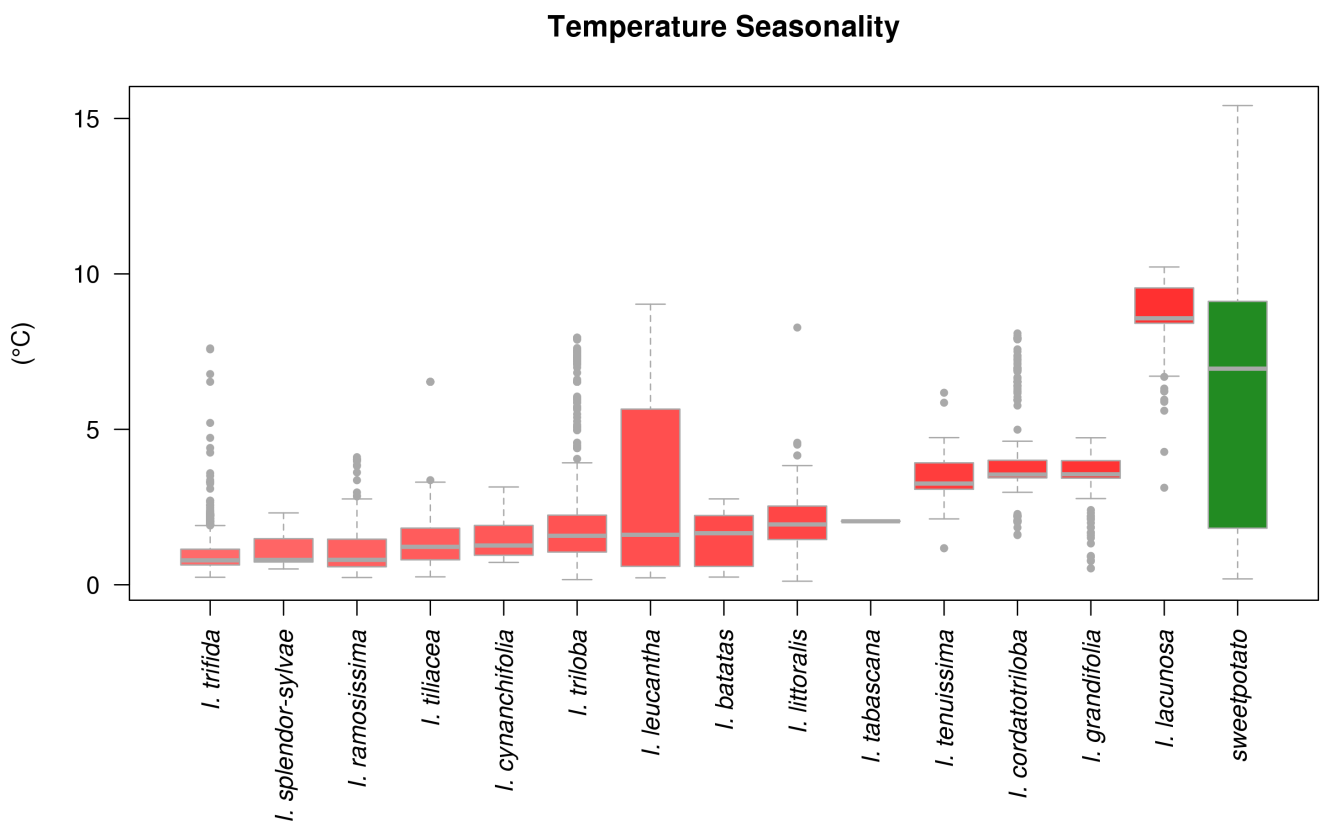


Figure S7E Climatic niches of CWR species and the sweetpotato crop for temperature seasonality.


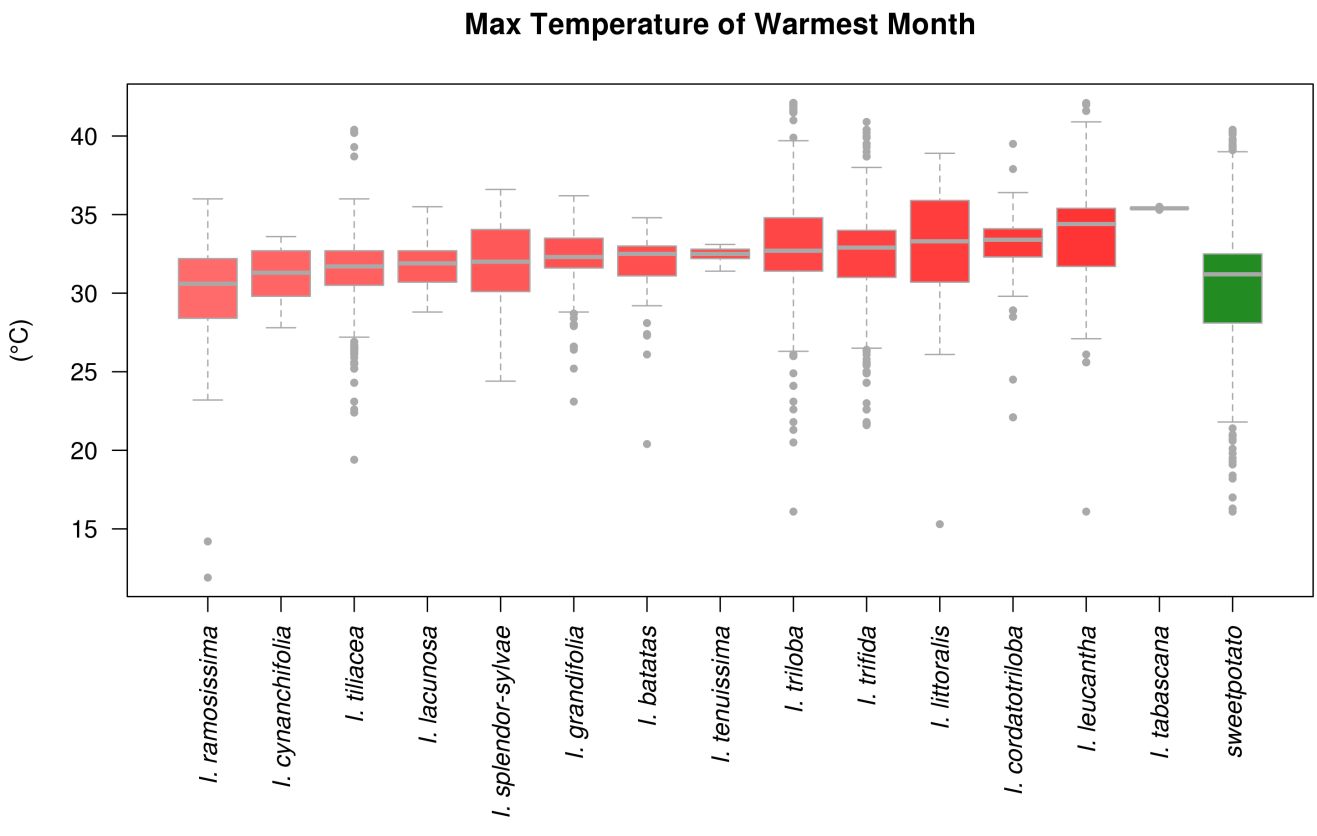


Figure S7F Climatic niches of CWR species and the sweetpotato crop for maximum temperature of the warmest month of the year.


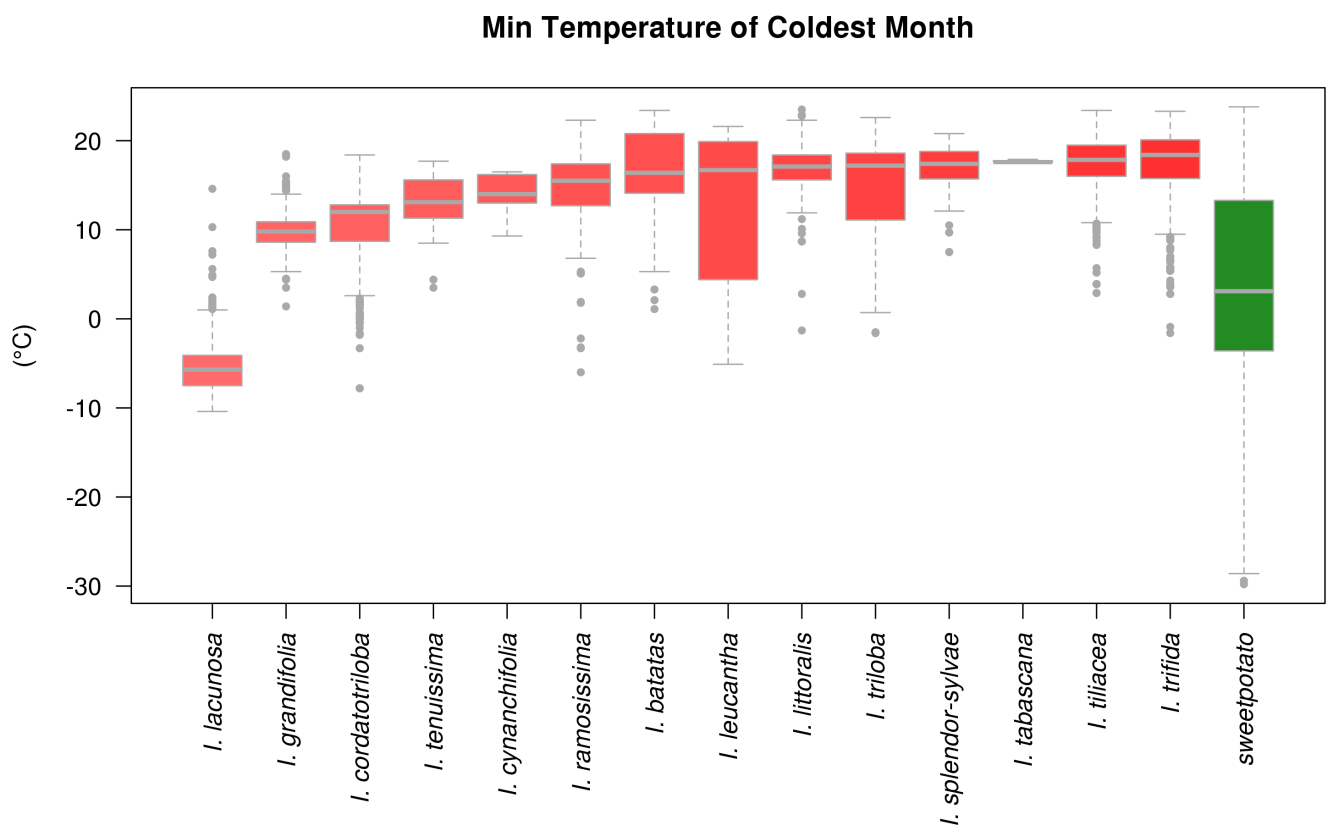


Figure S7G Climatic niches of CWR species and the sweetpotato crop for minimum temperature of the coldest month of the year.


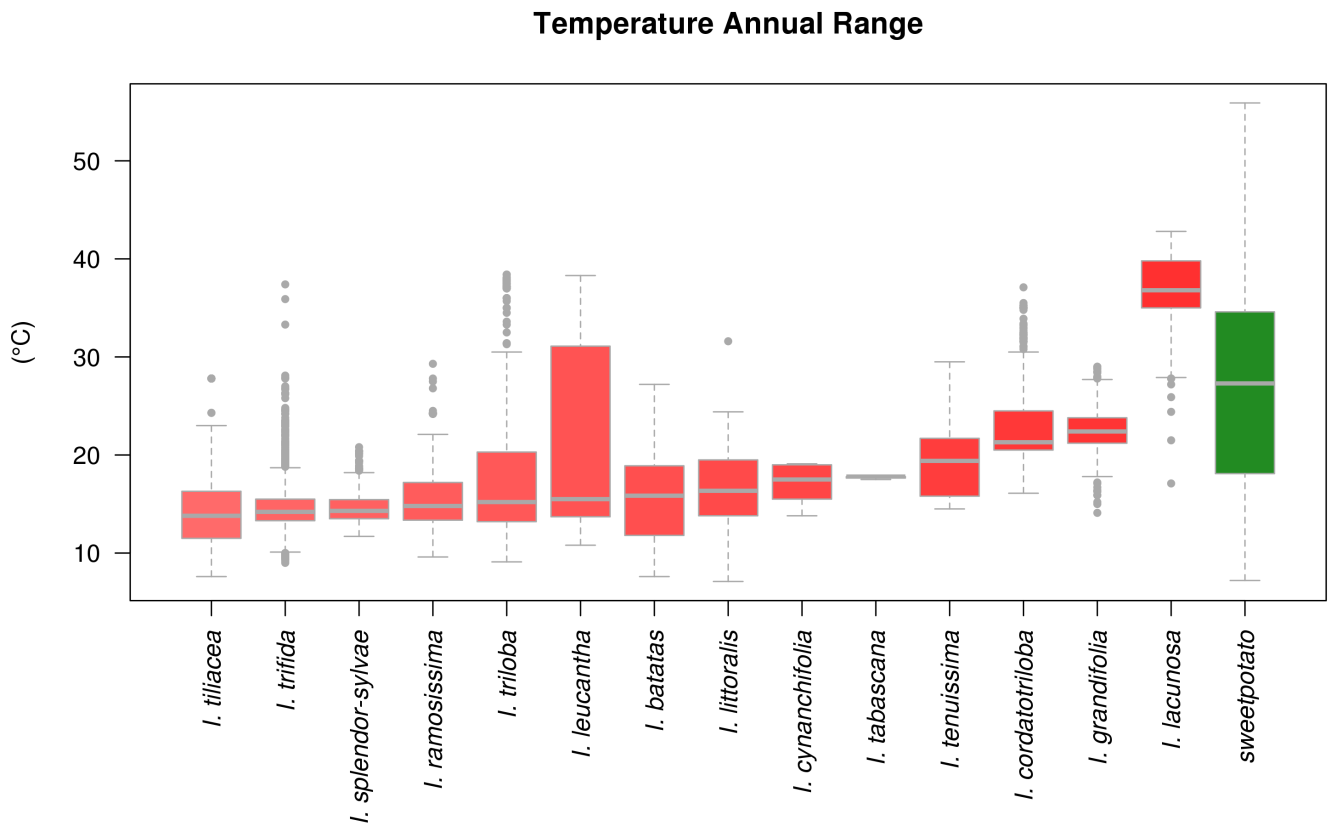


Figure S7H Climatic niches of CWR species and the sweetpotato crop for annual temperature range.


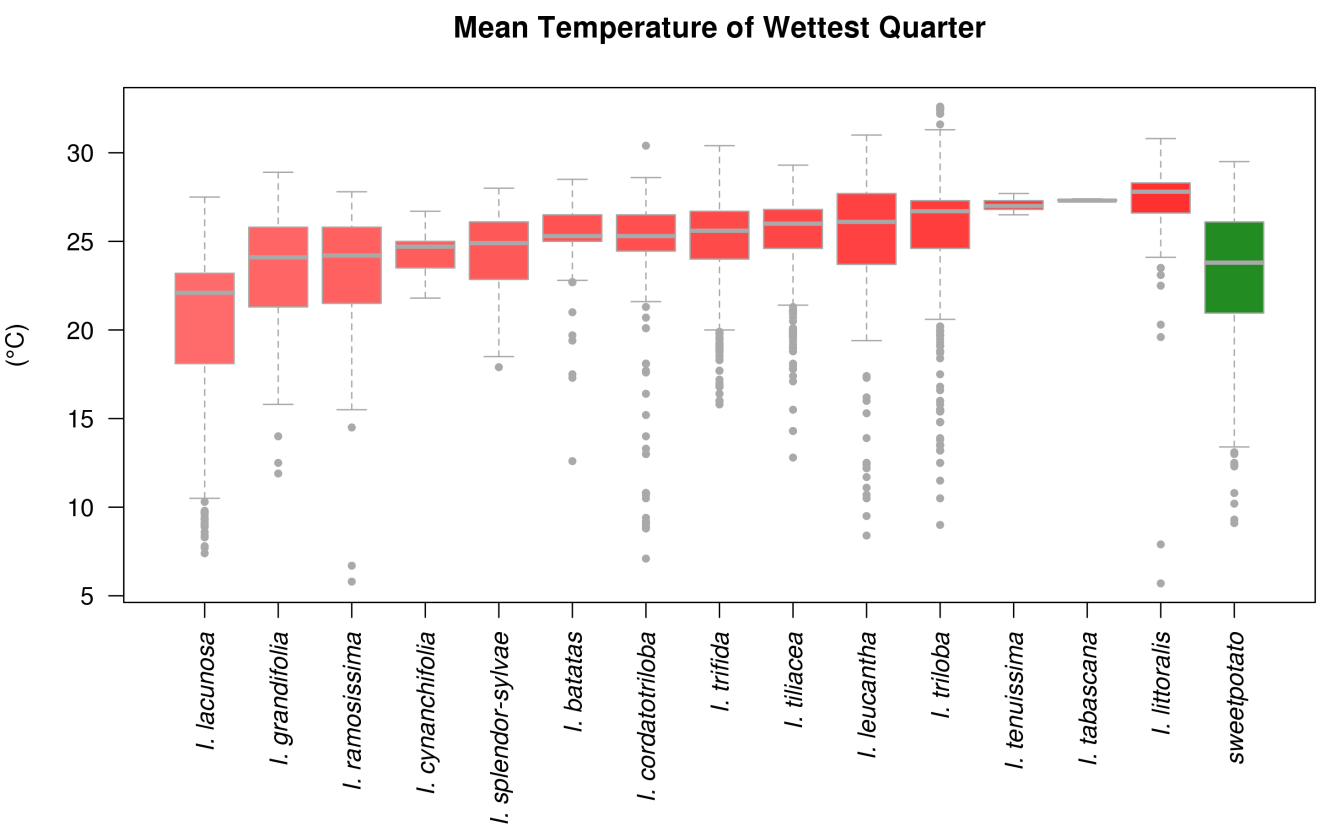


Figure S7I Climatic niches of CWR species and the sweetpotato crop for mean temperature of the wettest quarter of the year.


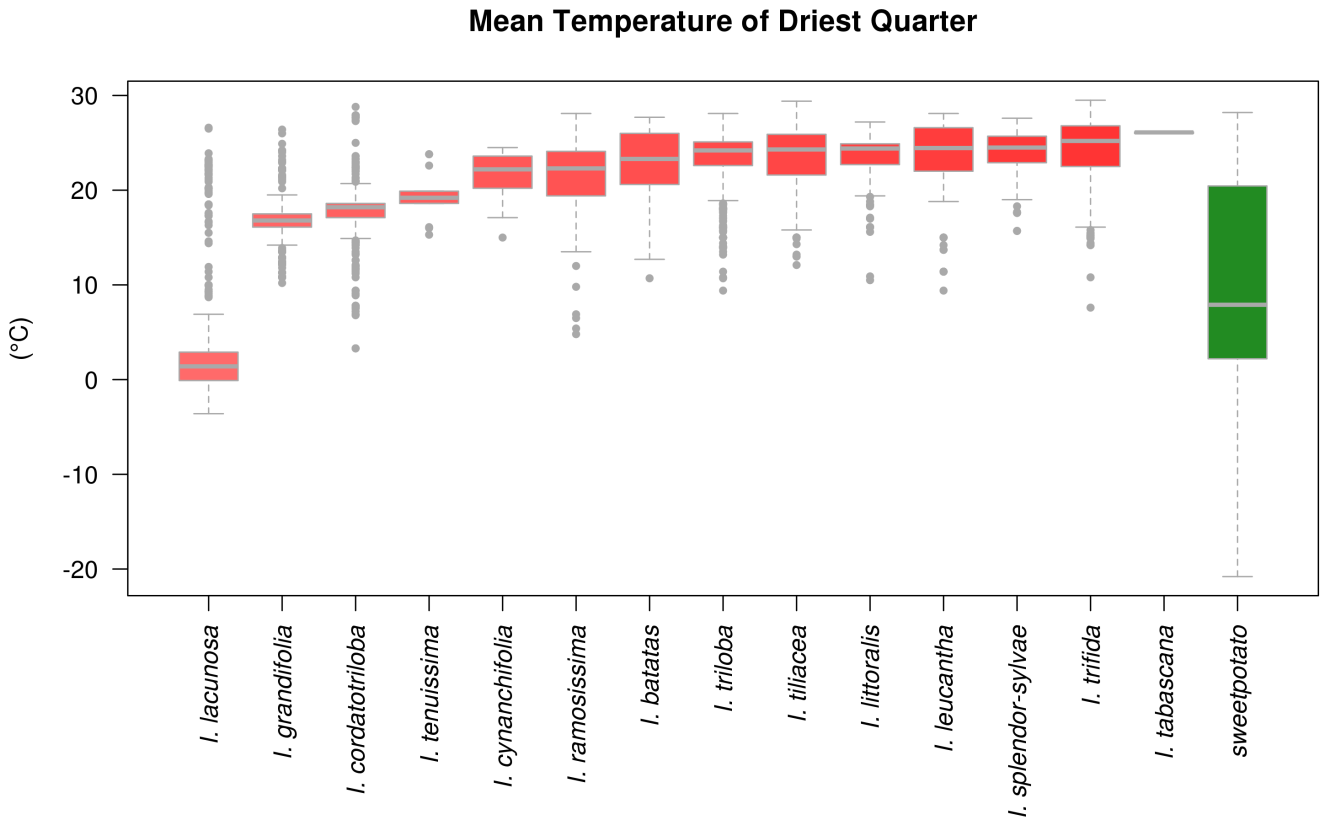


Figure S7J Climatic niches of CWR species and the sweetpotato crop for mean temperature of the driest quarter of the year.


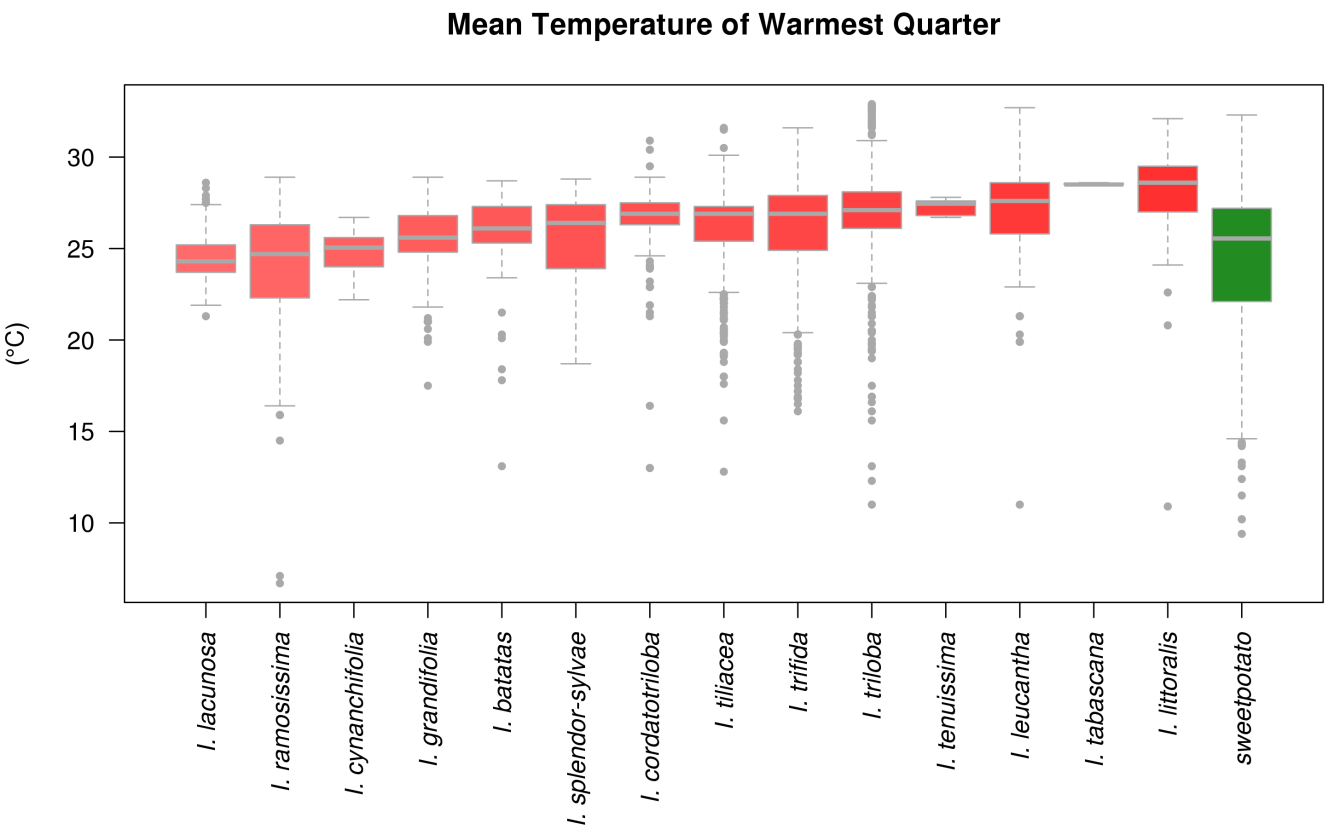


Figure S7K Climatic niches of CWR species and the sweetpotato crop for mean temperature of the warmest quarter of the year.


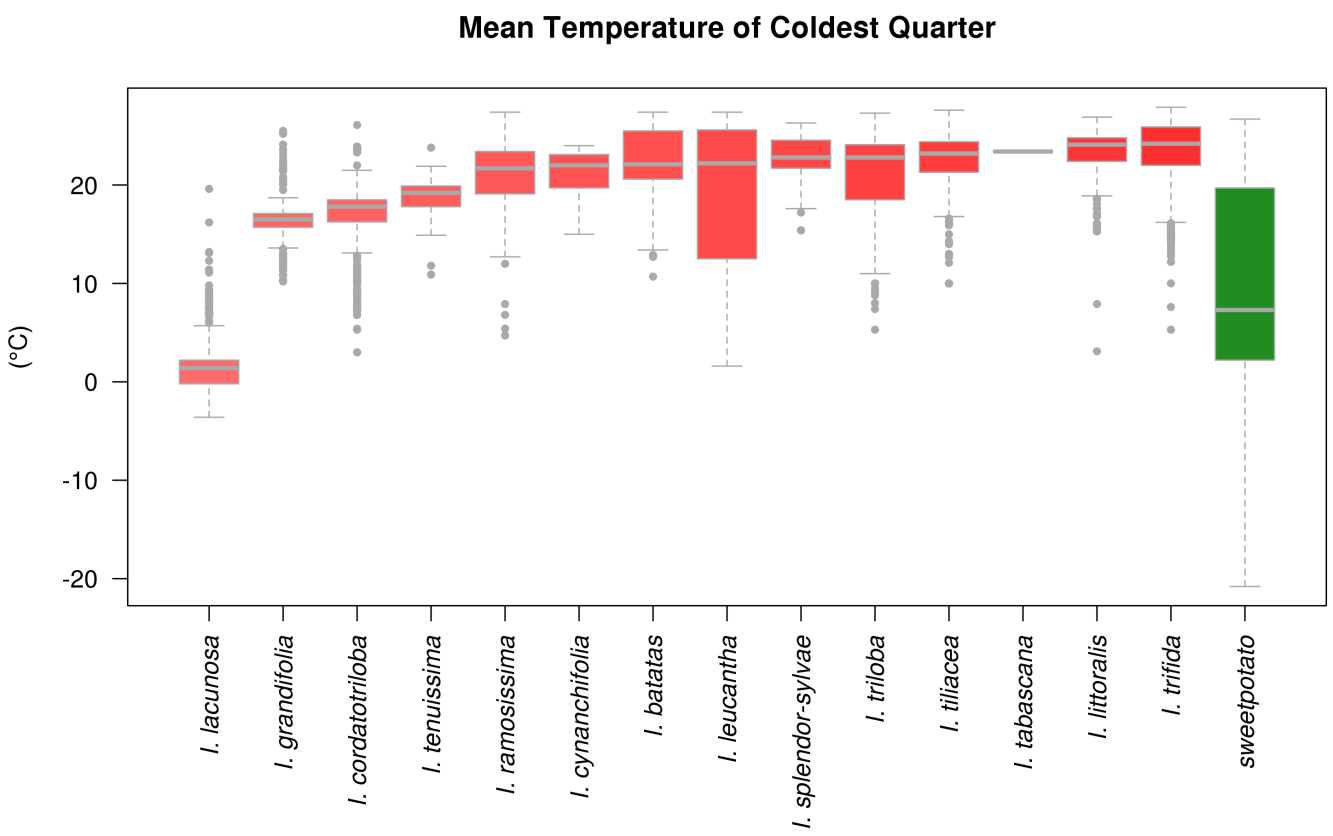


Figure S7L Climatic niches of CWR species and the sweetpotato crop for mean temperature of the coldest quarter of the year.


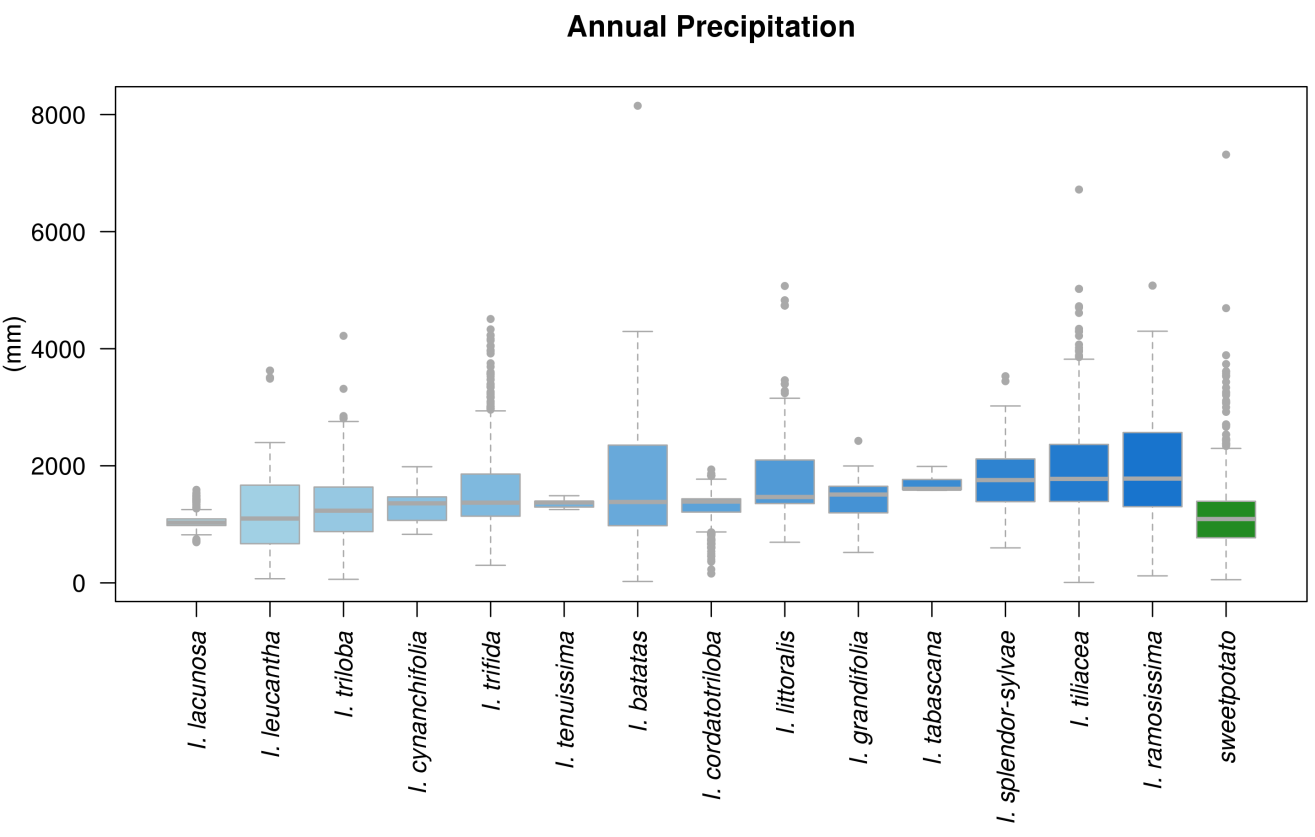


Figure S7M Climatic niches of CWR species and the sweetpotato crop for annual precipitation.


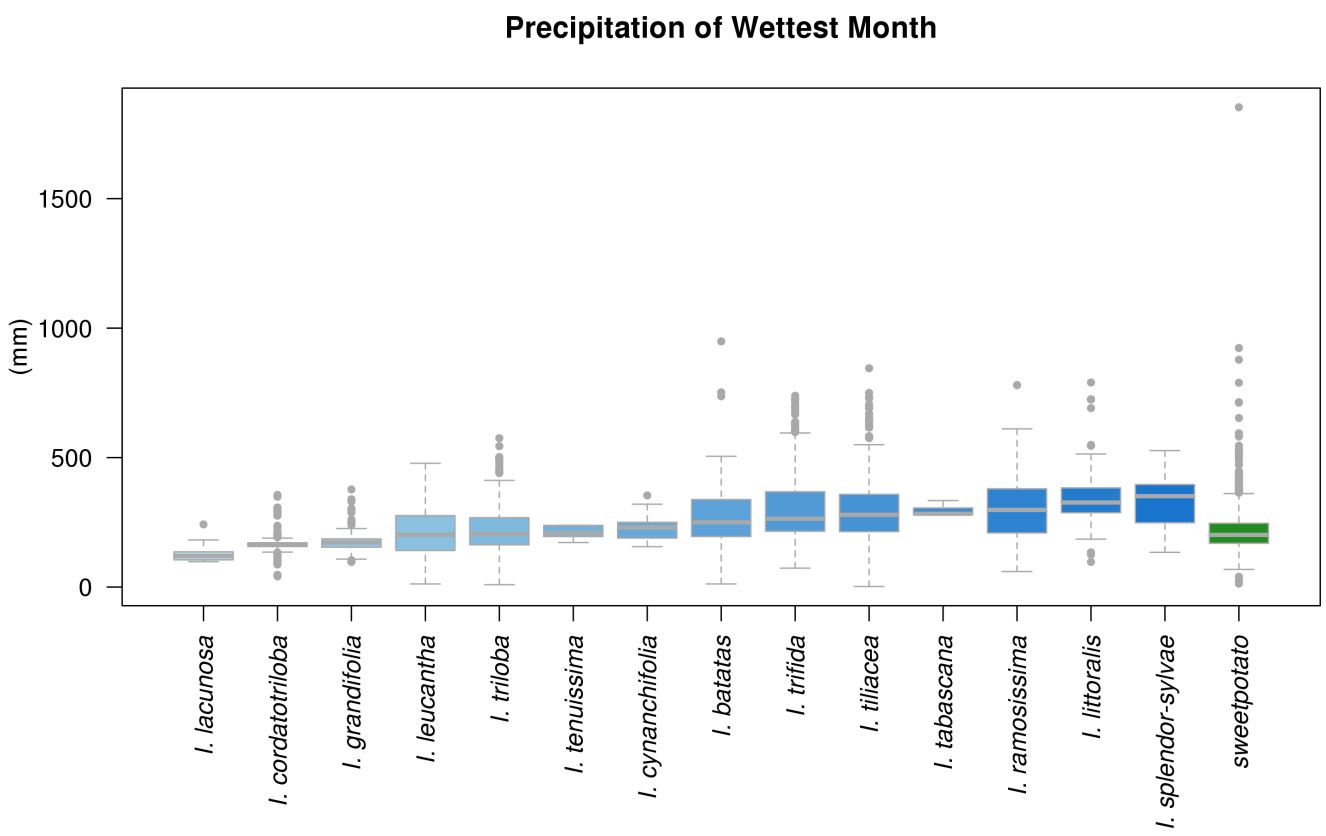


Figure S7N Climatic niches of CWR species and the sweetpotato crop for precipitation of the wettest month of the year.


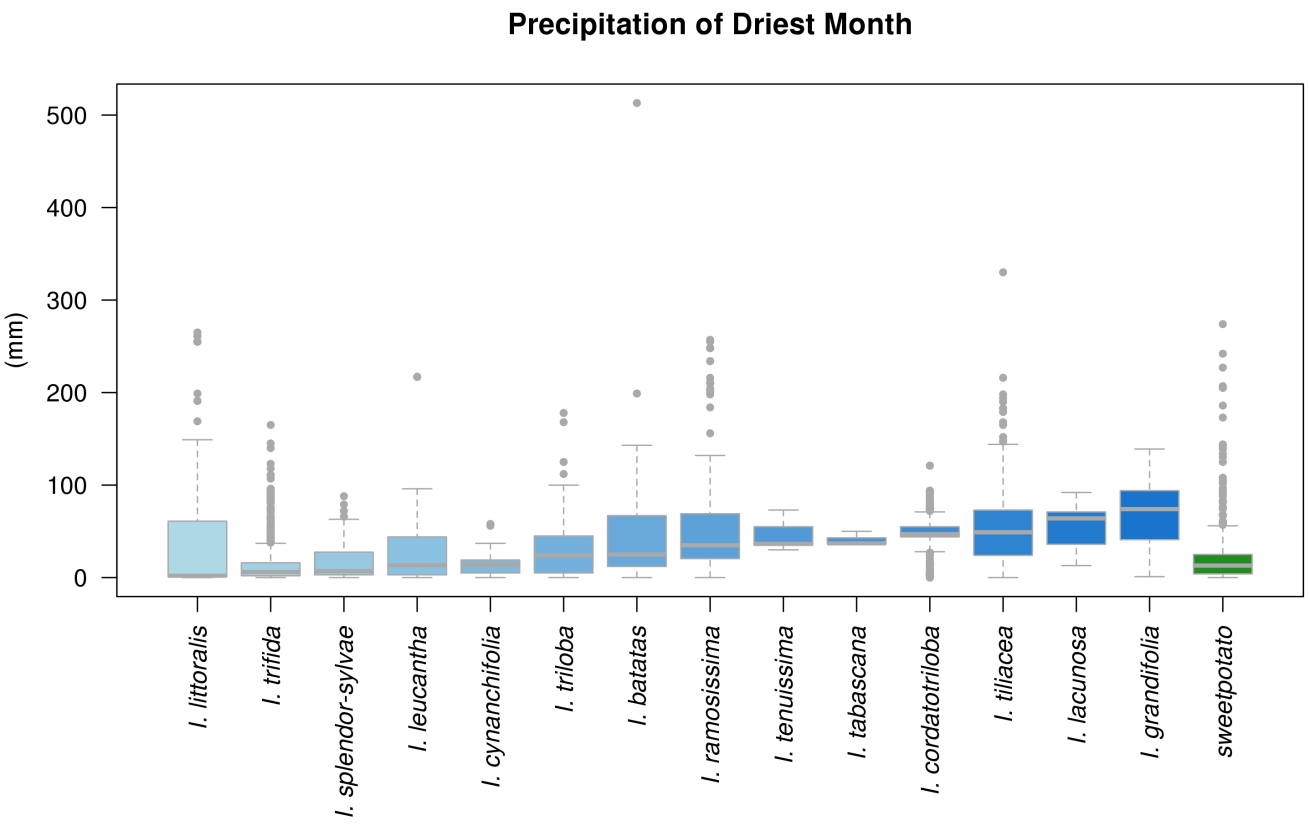


Figure S7O Climatic niches of CWR species and the sweetpotato crop for precipitation of the driest month of the year.


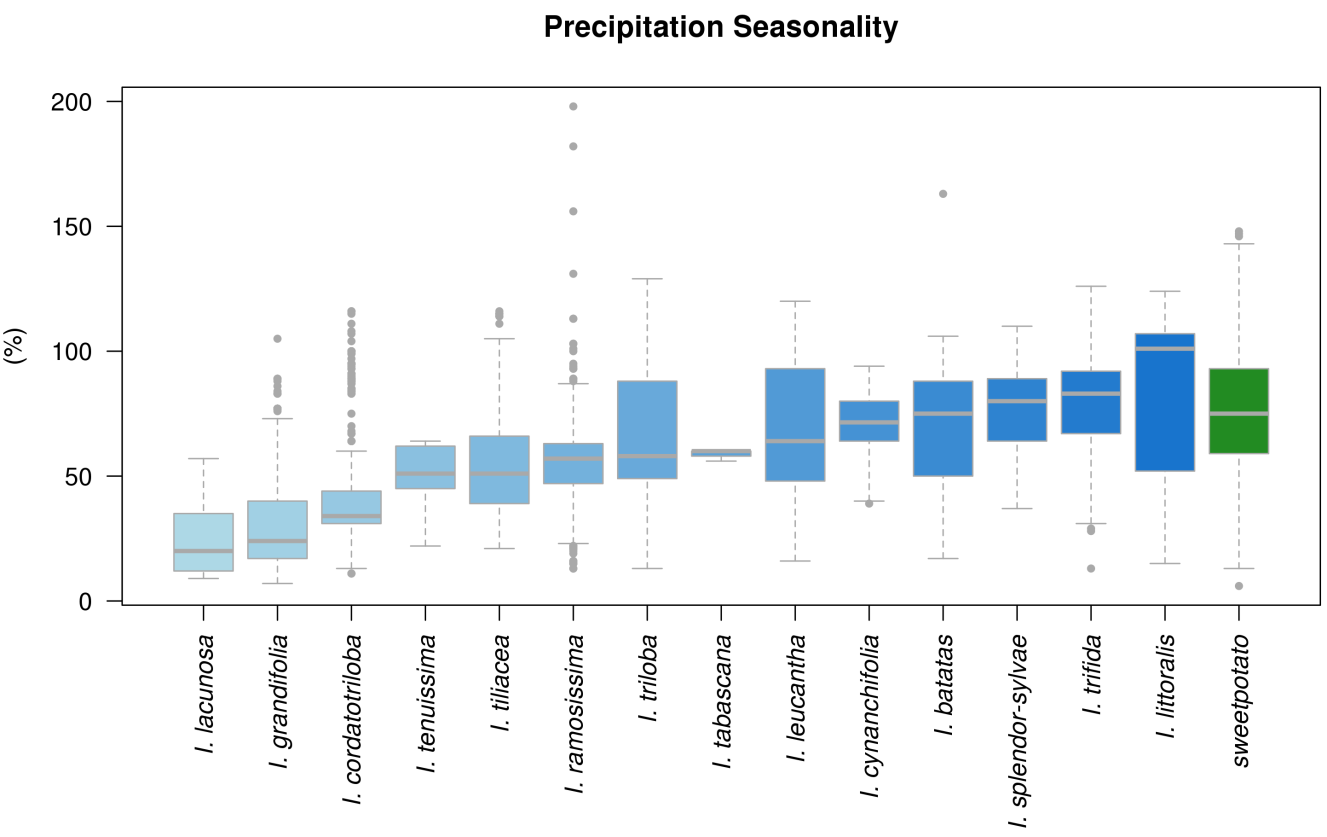


Figure S7P Climatic niches of CWR species and the sweetpotato crop for precipitation seasonality.


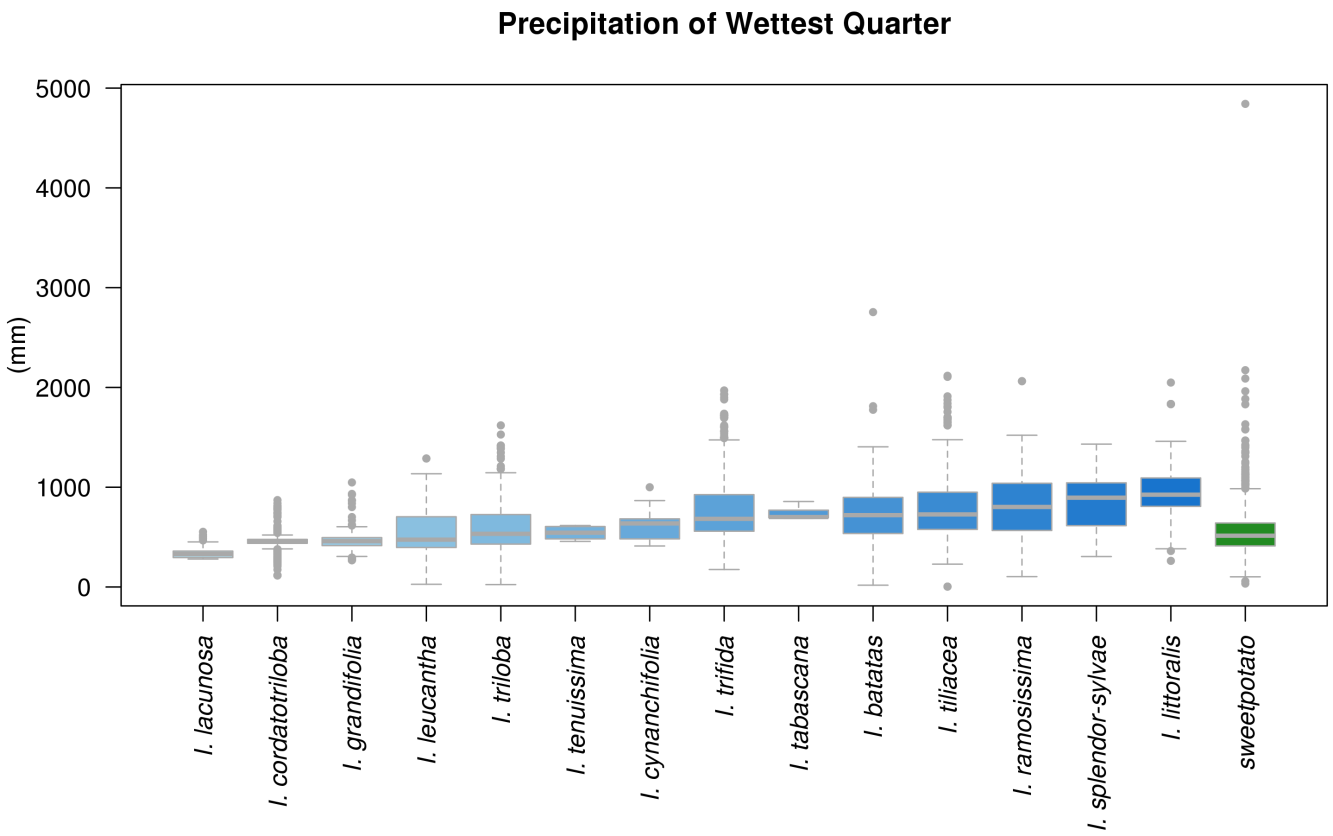


Figure S7Q Climatic niches of CWR species and the sweetpotato crop for precipitation of the wettest quarter of the year.


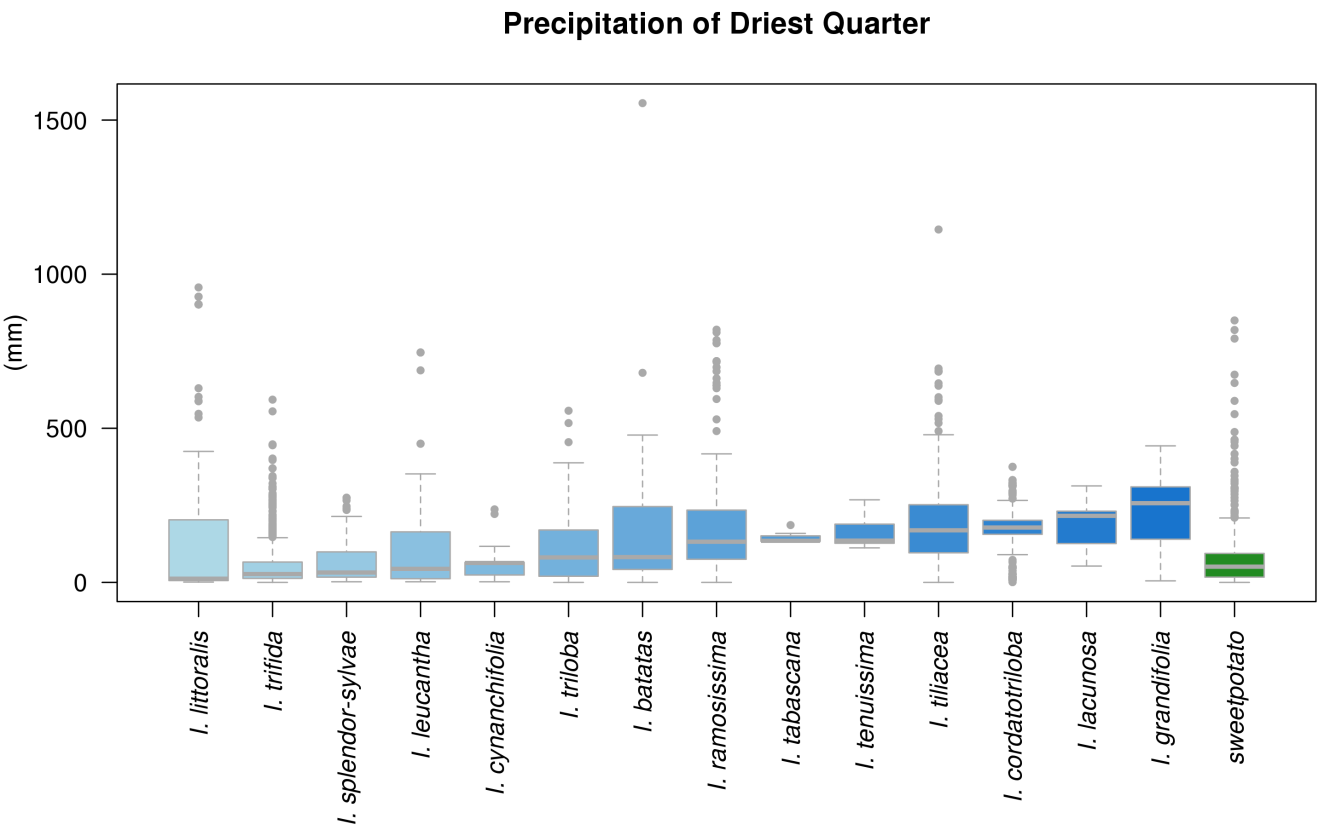


Figure S7R Climatic niches of CWR species and the sweetpotato crop for precipitation of the driest quarter of the year.


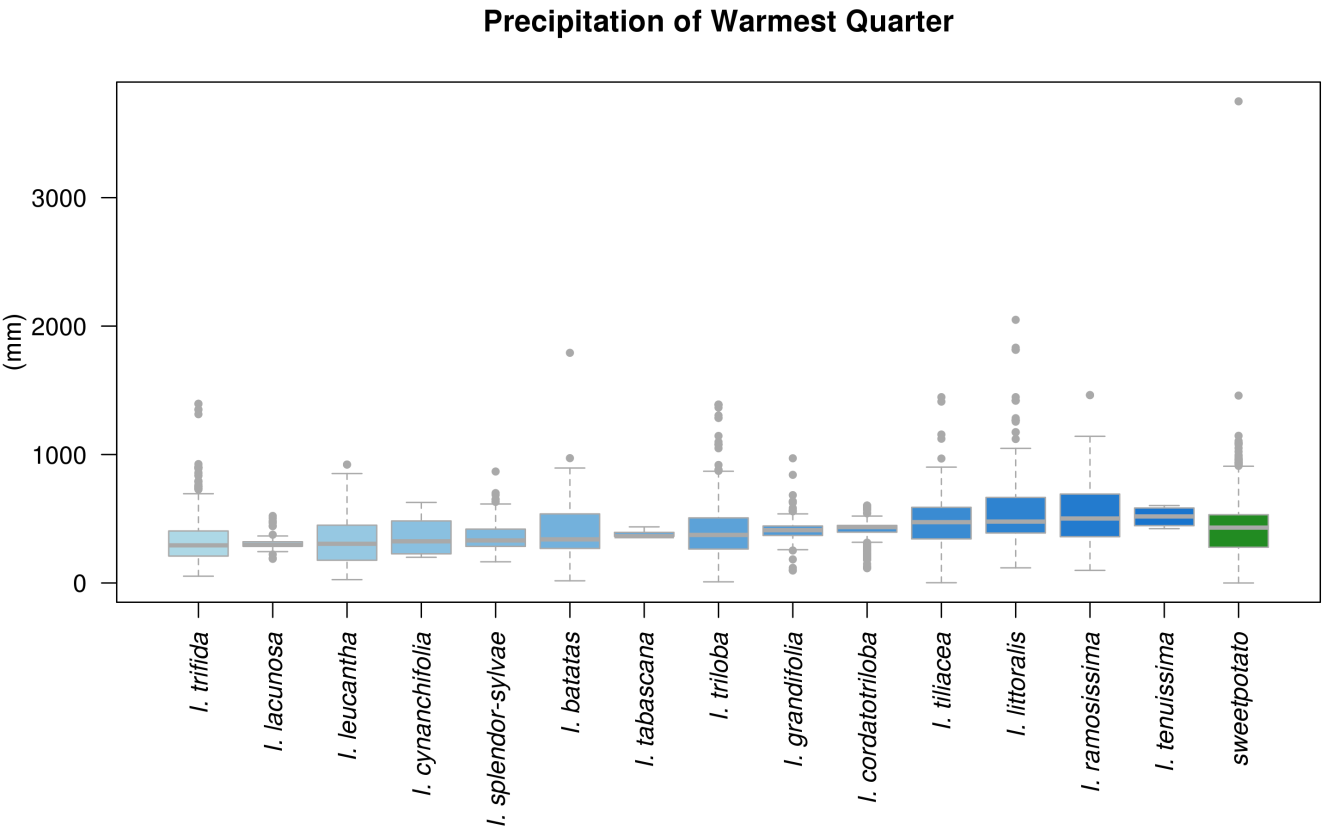


Figure S7S Climatic niches of CWR species and the sweetpotato crop for precipitation of the warmest quarter of the year.


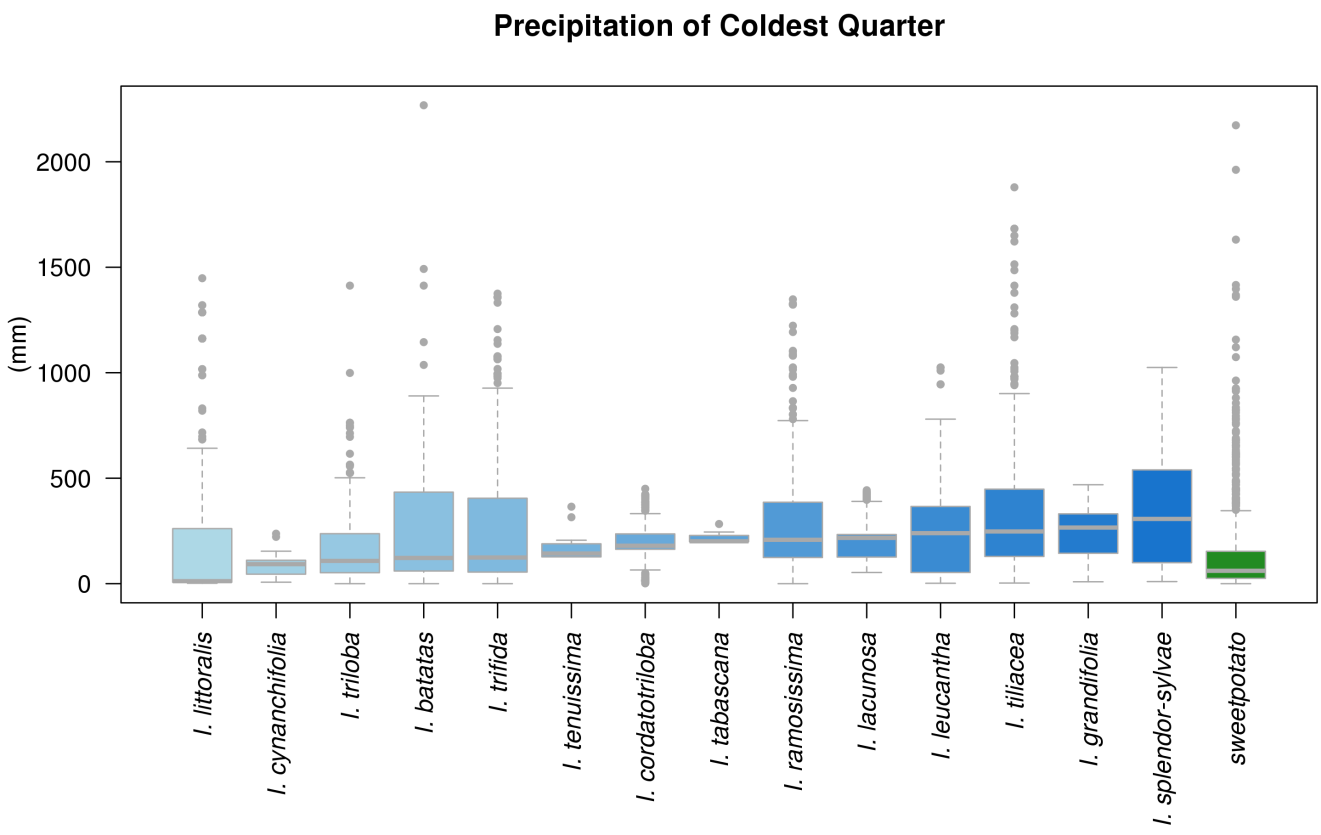


Figure S7T Climatic niches of CWR species and the sweetpotato crop for precipitation of the coldest quarter of the year.


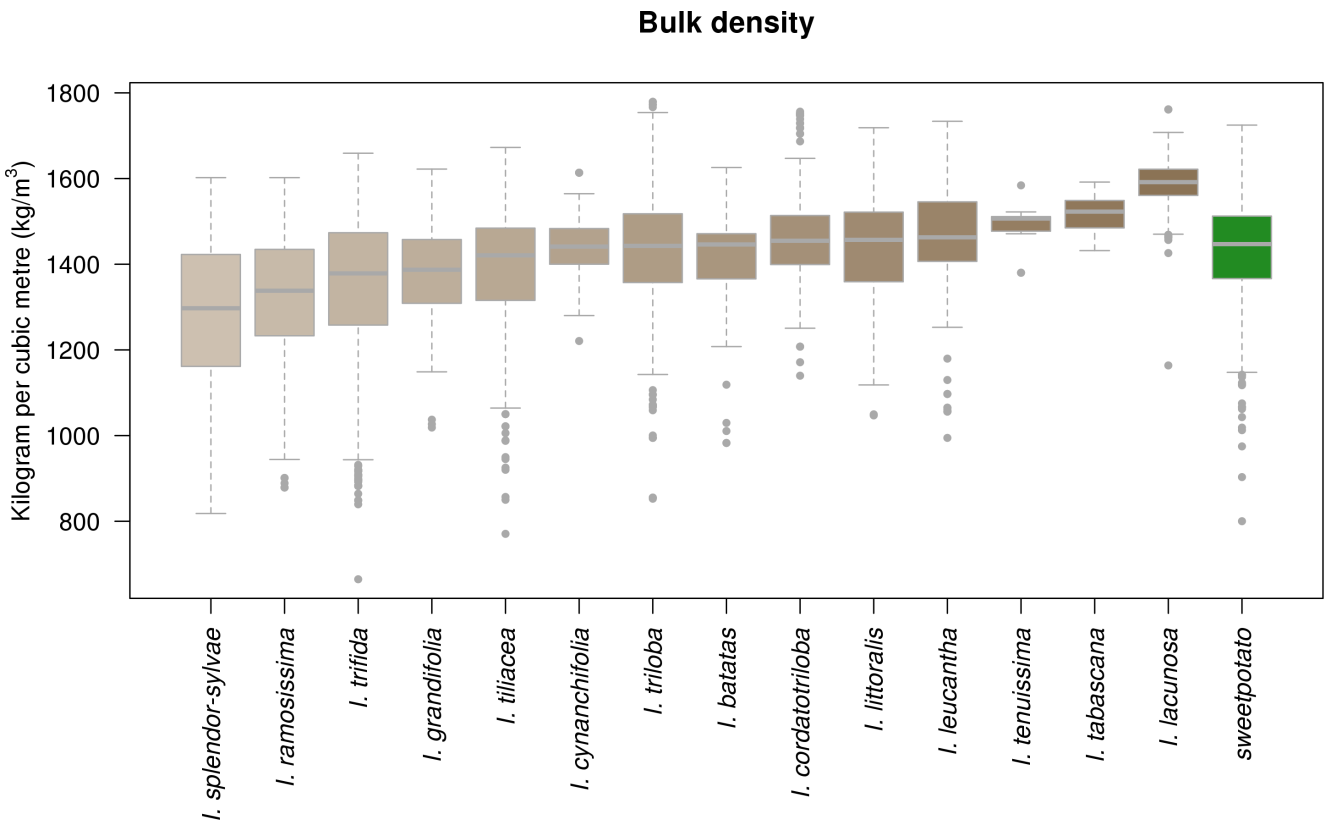


Figure S7U Edaphic niches of CWR species and the sweetpotato crop for bulk density.


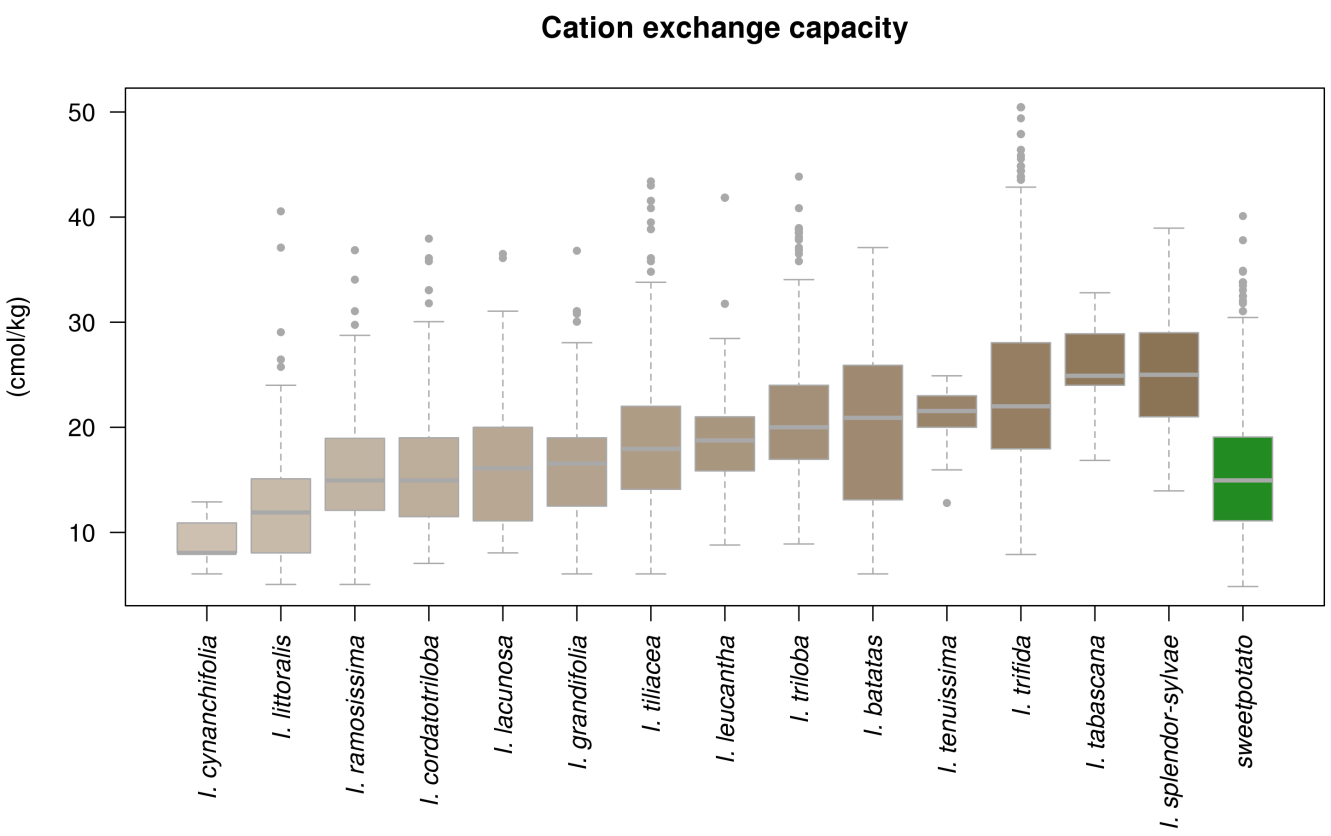


Figure S7V Edaphic niches of CWR species and the sweetpotato crop for cation exchange capacity.


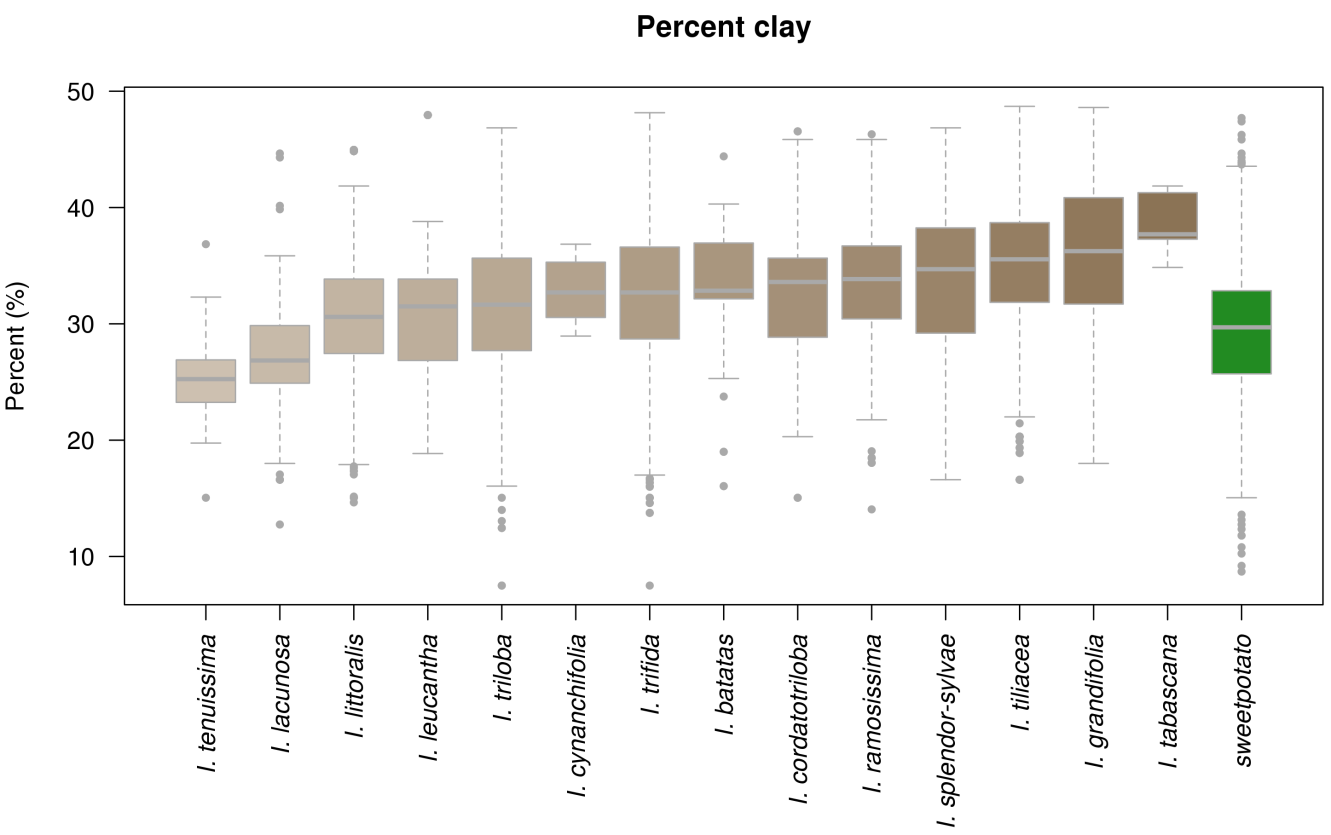


Figure S7W Edaphic niches of CWR species and the sweetpotato crop for percent clay.


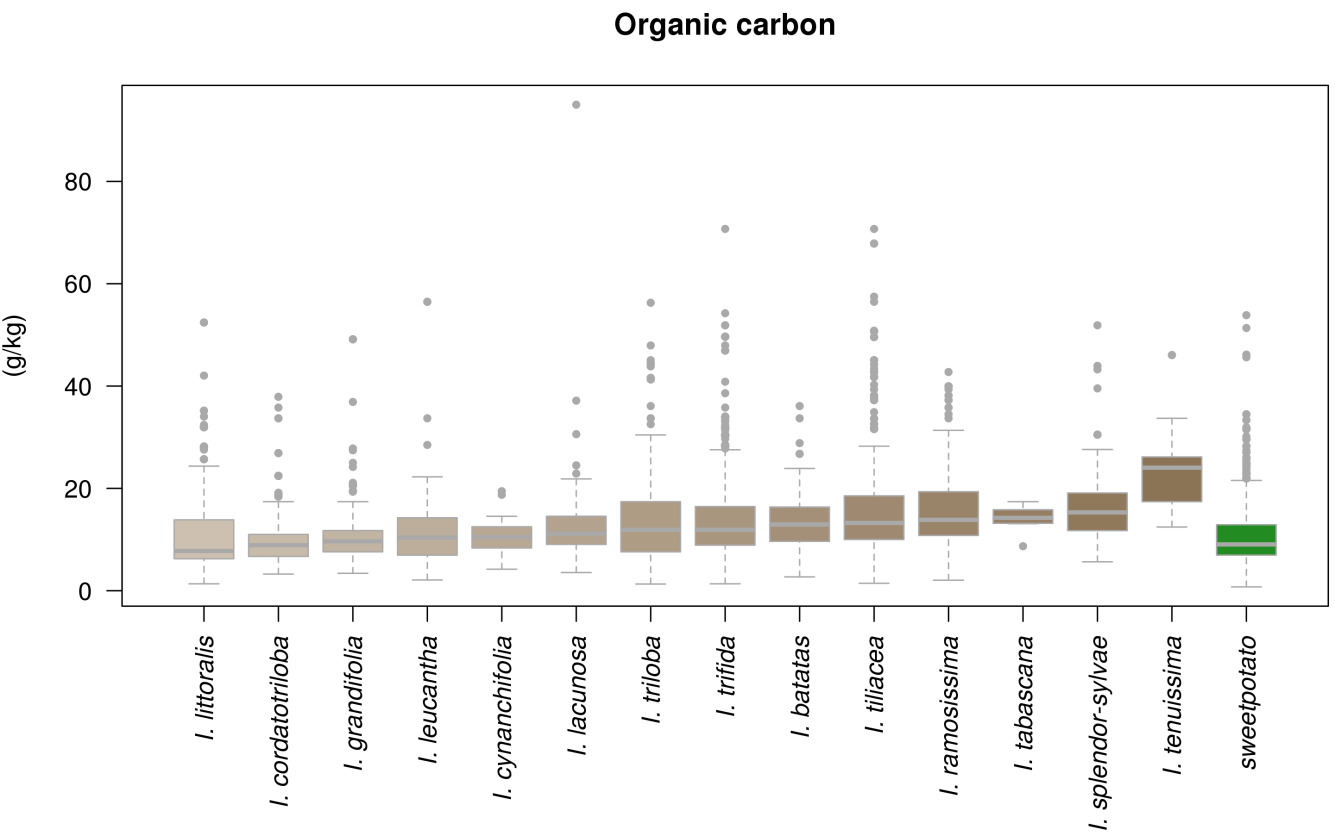


Figure S7X Edaphic niches of CWR species and the sweetpotato crop for organic carbon.


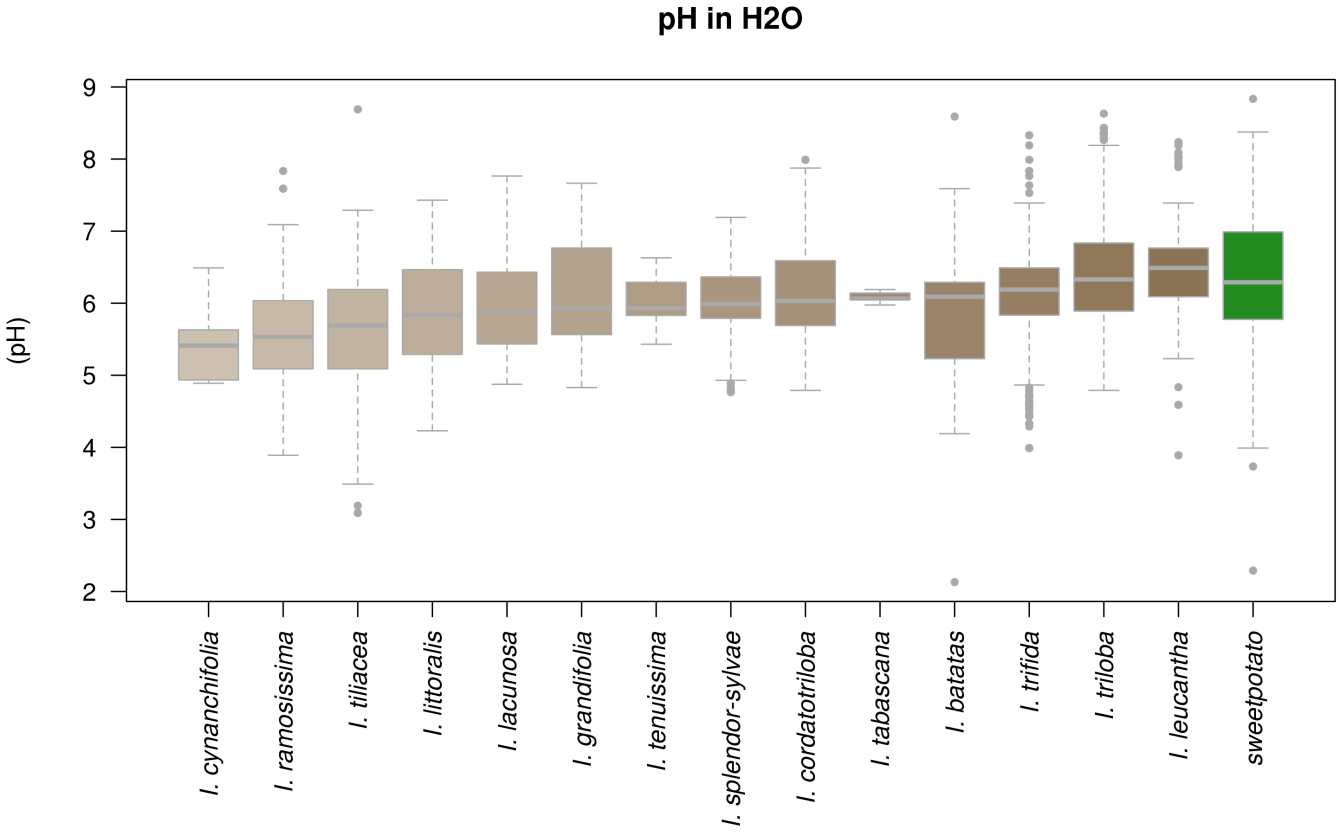


Figure S7Y Edaphic niches of CWR species and the sweetpotato crop for pH.


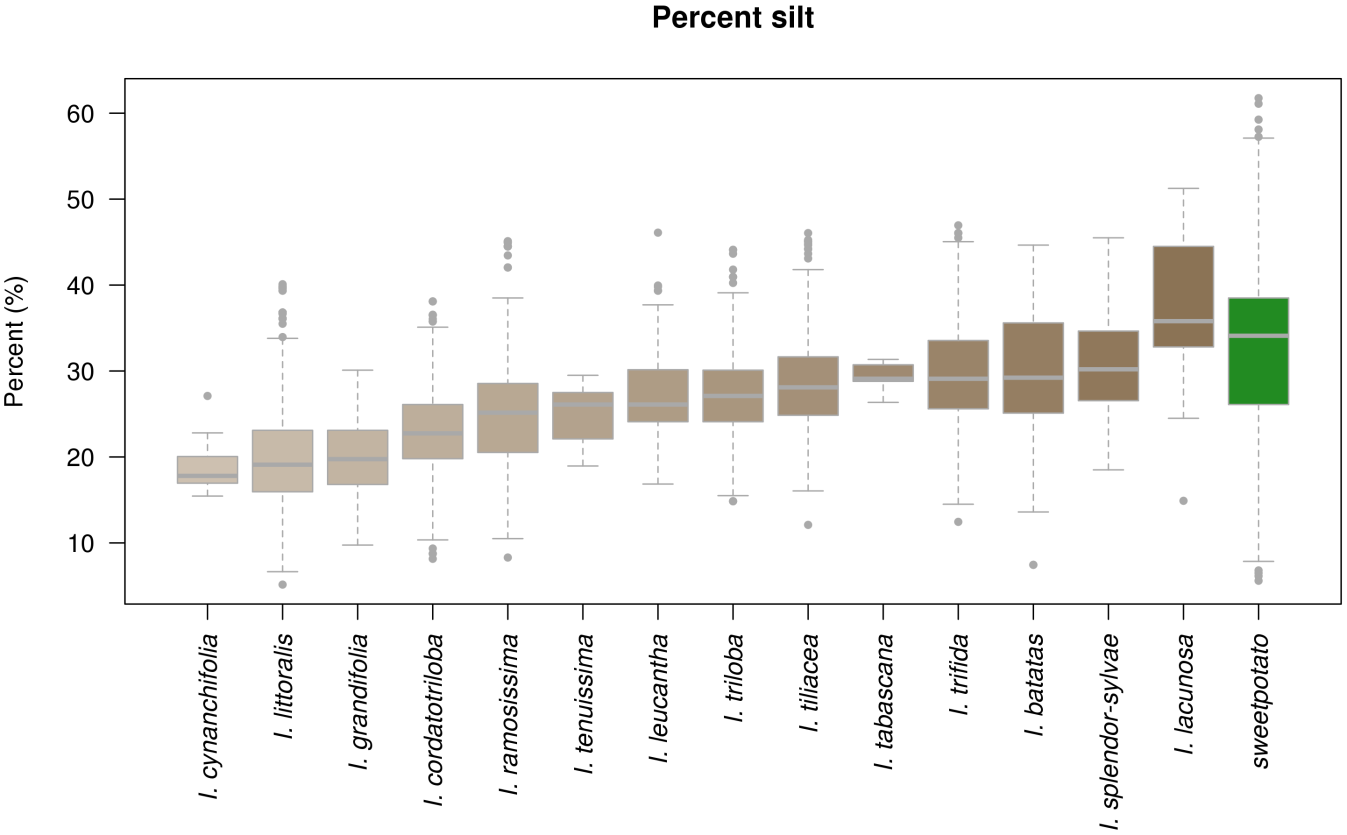


Figure S7Z Edaphic niches of CWR species and the sweetpotato crop for percent silt.


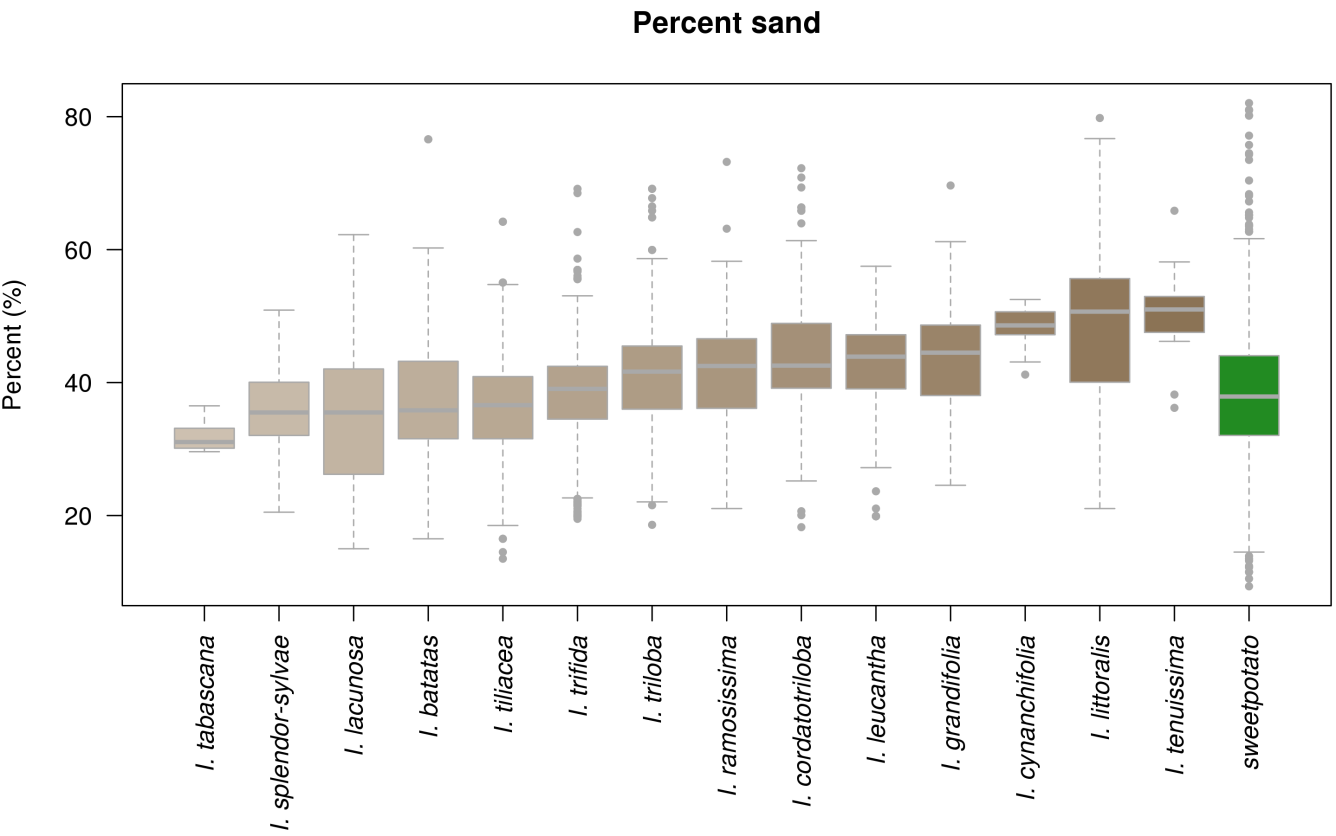


Figure S7AA Edaphic niches of CWR species and the sweetpotato crop for percent sand.

***Tables***

Table S1 Sources of occurrence data for assessed sweetpotato [*Ipomoea batatas* (L.) Lam.] crop wild relative species.

| Data provider | Record type | Number of records |
| --- | --- | --- |
| International Potato Center (CIP) | Germplasm accession | 585 |
| Leibniz Institute of Plant Genetics and Crop Plant Research | Germplasm accession | 1 |
| Millennium Seed Bank Partnership, Royal Botanic Gardens, Kew | Germplasm accession | 3 |
| USDA, National Plant Germplasm System, Germplasm Resources Information Network (USDA NPGS GRIN) | Germplasm accession | 160 |
| Atlas of Living Australia (Flora Atlas NT) (via GBIF) | Herbarium or other record | 356 |
| Australia's Virtual Herbarium (AD, BRI, CANB, CBG, DNA, HO, MEL, NSW, PERTH) | Herbarium or other record | 39 |
| Bioversity International | Herbarium or other record | 208 |
| Brazil Virtual Herbarium (CRIA) | Herbarium or other record | 61 |
| California Academy of Sciences Herbarium (CAS) | Herbarium or other record | 128 |
| Comisión Nacional para el Conocimiento y Uso de la Biodiversidad (CONABIO) (via GBIF) | Herbarium or other record | 97 |
| Consortium of Pacific Northwest Herbaria (CPNWH) | Herbarium or other record | 5 |
| DIVEA, DEP, FEEMA Herbário Alberto Castellanos (GUA) | Herbarium or other record | 18 |
| Fairchild Tropical Botanical Garden (FTG) (via GBIF) | Herbarium or other record | 712 |
| Florida State University Robert K. Godfrey Herbarium (FSU) | Herbarium or other record | 6 |
| Harvard University Herbarium (HUH) | Herbarium or other record | 8 |
| Herbarium of National Taiwan University (TAI) (via GBIF) | Herbarium or other record | 63 |
| Herbarium of Taiwan Forestry Research Institute (TAIF) (via GBIF) | Herbarium or other record | 56 |
| Instituto Nacional de Biodiversidad, Costa Rica (INB) (via GBIF) | Herbarium or other record | 87 |
| International Center for Tropical Agriculture (CIAT)- Bioversity International, USDA. Atlas of Paraguayan Crop Wild Relatives. | Herbarium or other record | 155 |
| International Center for Tropical Agriculture (CIAT)- C. Azurdia, K.A. Williams, D.E. Williams, V. Van Damme, A. Jarvis and S.E. Castaño. 2011. Atlas of Guatemalan Crop Wild Relatives. | Herbarium or other record | 139 |
| International Potato Center (CIP) | Herbarium or other record | 231 |
| Jardim Botanico de Rio de Janeiro (JABOT/R) | Herbarium or other record | 106 |
| Jardim Botânico do Rio de Janeiro (JBRJ) (via GBIF) | Herbarium or other record | 119 |
| Louisiana State University Herbarium (LSU) (via GBIF) | Herbarium or other record | 116 |
| Manchester University Herbarium (MANCH) | Herbarium or other record | 2 |
| McDonald & Austin (1990) | Herbarium or other record | 1 |
| Missouri Botanical Garden Herbarium (MO) | Herbarium or other record | 44 |
| Museo Nacional de Costa Rica (MNCR) (via GBIF) | Herbarium or other record | 80 |
| Museum national d'Histoire naturelle (MNHN) (via GBIF) | Herbarium or other record | 509 |
| Nationaal Herbarium Nederland (NHN) | Herbarium or other record | 112 |
| Natural History Museum UK Herbarium (BM) | Herbarium or other record | 19 |
| New York Botanical Garden Herbarium (NY) | Herbarium or other record | 454 |
| Plants of Taiwan | Herbarium or other record | 56 |
| Real Jardín Botánico de Madrid (MA) | Herbarium or other record | 25 |
| Royal Botanic Gardens, Kew (K) | Herbarium or other record | 20 |
| Scotland & Wood, personal communication | Herbarium or other record | 8 |
| Smithsonian Institution, National Herbarium (US) | Herbarium or other record | 357 |
| The Field Museum (F) | Herbarium or other record | 58 |
| Universidad del Valle Colombia Herbarium (CUVC) | Herbarium or other record | 4 |
| University of California Jepson Herbarium (UC/Jeps) | Herbarium or other record | 63 |
| University of California Riverside Herbarium (UCR) | Herbarium or other record | 23 |
| University of Kansas McGregor Herbarium (KU) (via GBIF) | Herbarium or other record | 79 |
| University of Oklahoma, Robert Bebb Herbarium (OKL) (via GBIF) | Herbarium or other record | 53 |
| US Academy of Natural Sciences Philadelphia Herbarium (PH) | Herbarium or other record | 45 |
| USDA, National Plant Germplasm System, Germplasm Resources Information Network (USDA NPGS GRIN) | Herbarium or other record | 131 |
| West Virginia University Herbarium (WVU) | Herbarium or other record | 12 |

Table S2 Ecogeographic variables utilized in sweetpotato [*Ipomoea batatas* (L.) Lam.] crop wild relative potential distribution modeling and climatic niche analyses

| Variable number | Variable name | Units |
| --- | --- | --- |
| 0 | Altitude | m |
| 1 | Annual mean temperature | ºC |
| 2 | Mean diurnal temperature range | ºC |
| 3 | Isothermality | N/A |
| 4 | Temperature seasonality (standard deviation) | ºC |
| 5 | Maximum temperature of warmest month | ºC |
| 6 | Minimum temperature of coldest month | ºC |
| 7 | Temperature annual range | ºC |
| 8 | Mean temperature of wettest quarter | ºC |
| 9 | Mean temperature of driest quarter | ºC |
| 10 | Mean temperature of warmest quarter | ºC |
| 11 | Mean temperature of coldest quarter | ºC |
| 12 | Annual precipitation | mm |
| 13 | Precipitation of wettest month | mm |
| 14 | Precipitation of driest month | mm |
| 15 | Precipitation seasonality (coefficient of variation) | % |
| 16 | Precipitation of wettest quarter | mm |
| 17 | Precipitation of driest quarter | mm |
| 18 | Precipitation of warmest quarter | mm |
| 19 | Precipitation of coldest quarter | mm |
| 20 | Bulk density | kg/m^3^ |
| 21 | Cation exchange capacity | cmol/kg |
| 22 | Percent clay | % |
| 23 | Organic carbon | g/kg |
| 24 | pH in H_2_O | pH |
| 25 | Percent silt | % |
| 26 | Percent sand | % |

Table S3 Gap analysis and comparable expert evaluation priorities results per sweetpotato [*Ipomoea batatas* (L.) Lam.] crop wild relative.

| Taxon | Total Records | Total Records- Distinct | H Records- Total | H Records- Distinct | G Records- Total | G Records- Distinct | SRS | Ecogeographic Variables | ATAUC | STAUC | ASD15 | cAUC | GRS | ERS | FPS | FPCAT | EPS 1 | EPS 2 | EPS 3 | EPS 4 | EPS 5 | EPS mean |
| --- | --- | --- | --- | --- | --- | --- | --- | --- | --- | --- | --- | --- | --- | --- | --- | --- | --- | --- | --- | --- | --- | --- |
| *I. batatas* | 104 | 44 | 100 | 44 | 4 | 0 | 0.38 | 2, 4, 8, 13, 14, 19, 24 | 0.78 | 0.06 | 11.19 | 0.28 | 0.00 | 0.00 | 0.13 | HPS | 1 | 1 | 7 | 1 | 2 | 2.4 |
| *I. cordatotriloba* | 424 | 290 | 321 | 229 | 103 | 67 | 2.43 | 1, 2, 3, 9, 13, 15, 18 | 0.97 | 0.01 | 0.93 | 0.47 | 1.24 | 2.86 | 2.18 | HPS | 6 | 7 | 5 | 1 | 1 | 4 |
| *I. cynanchifolia* | 37 | 17 | 36 | 17 | 1 | 0 | 0.27 | 0, 15, 16, 18, 19, 22, 23, 26(*) | 0.76 | NA | NA | 0.26 | 0.00 | 0.00 | 0.09 | HPS | 0 | 7 | 2 | NA | 0 | 2.3 |
| *I. grandifolia* | 460 | 334 | 336 | 254 | 124 | 83 | 2.70 | 3, 7, 8, 9, 10, 15, 16 | 0.95 | 0.01 | 0.02 | 0.45 | 2.54 | 7.22 | 4.15 | MPS | 3 | 3 | 5 | 1 | 1 | 2.6 |
| *I. lacunosa* | 413 | 252 | 403 | 251 | 10 | 1 | 0.24 | 0, 3, 5, 7, 9, 12, 13, 25 | 0.97 | 0.00 | 0.49 | 0.47 | 0.07 | 0.91 | 0.41 | HPS | 4 | 8 | 2 | 1 | 1 | 3.2 |
| *I. leucantha* | 134 | 93 | 116 | 79 | 18 | 15 | 1.34 | 1, 2, 3, 8, 12, 16, 20, 24 | 0.91 | 0.04 | 0.19 | 0.41 | 0.06 | 0.57 | 0.66 | HPS | 4 | 7 | 3 | NA | 2 | 4 |
| *I. littoralis* | 468 | 340 | 466 | 338 | 2 | 2 | 0.04 | 1, 2, 4, 15, 18, 19, 20, 23 | 0.98 | 0.00 | 0.62 | 0.48 | 0.02 | 0.07 | 0.05 | HPS | 0 | 3 | 2 | 0 | 0 | 1 |
| *I. ramosissima* | 341 | 217 | 307 | 187 | 34 | 30 | 1.00 | 0, 7, 11, 13, 19, 20, 23, 24 | 0.85 | 0.02 | 1.07 | 0.35 | 0.50 | 2.34 | 1.28 | HPS | 4 | 3 | 4 | 1 | 2 | 2.8 |
| *I. splendor-sylvae* | 177 | 135 | 161 | 130 | 16 | 9 | 0.90 | 0, 5, 11, 19, 20 | 0.90 | 0.04 | 2.76 | 0.40 | 0.97 | 4.88 | 2.25 | HPS | 1 | 7 | 3 | 1 | 0 | 2.4 |
| *I. tabascana* | 8 | 6 | 4 | 4 | 4 | 2 | 5.00 | 22, 23, 24, 26(*) | 0.87 | NA | NA | 0.37 | 10.00 | 4.29 | 6.43 | LPS | 0 | 1 | 2 | 0 | 0 | 0.6 |
| *I. tenuissima* | 34 | 12 | 31 | 12 | 3 | 1 | 0.88 | 10, 13, 14, 21(*) | 0.95 | NA | NA | 0.45 | 0.32 | 1.05 | 0.75 | HPS | 0 | 5 | 3 | 0 | 0 | 1.6 |
| *I. tiliacea* | 740 | 441 | 679 | 400 | 61 | 44 | 0.82 | 0, 5, 11, 12, 17, 19, 20, 24 | 0.94 | 0.01 | 0.37 | 0.44 | 0.59 | 1.58 | 1.00 | HPS | 4 | 1 | 5 | 1 | 1 | 2.4 |
| *I. trifida* | 1409 | 1023 | 1161 | 888 | 248 | 159 | 1.76 | 0, 5, 11, 12, 13, 24 | 0.92 | 0.01 | 0.01 | 0.42 | 2.24 | 4.95 | 2.98 | MPS | 3 | 1 | 4 | 4 | 6 | 3.6 |
| *I. triloba* | 865 | 446 | 744 | 376 | 121 | 74 | 1.40 | 0, 2, 3, 9, 12, 16, 24 | 0.93 | 0.01 | 0.12 | 0.43 | 1.07 | 4.12 | 2.20 | HPS | 6 | 1 | 4 | 4 | 6 | 4.2 |

Distinct records denote occurrence records with unique coordinates. H records denote herbarium and other locality data used to inform species distribution models. G records denote germplasm accessions accessible to the global community. Ecogeographic variables lists those species-specific bioclimatic and edaphic variables used in Maxent modeling per taxon (Table S2); (*) denotes species for which an ensemble model was derived. Maxent assessment techniques include the 5-fold average area under the ROC curve of test data (ATAUC), the standard deviation of the test AUC of the 5 different folds (STAUC), the proportion of the potential distribution coverage with standard deviation above 0.15 (ASD15), and the calibrated AUC ( cAUC). The final priority score (FPS) for the species is the mean of the sampling representativeness score (SRS), geographic representativeness score (GRS), and ecological representativeness score (ERS). Final priority categories (FPCAT) for the CWR of sweetpotato included high (HPS), medium (MPS), and low (LPS) priority species for further collecting for *ex situ* conservation. EPS denotes comparable expert priority scores for 5 experts, along with their mean value.

Table S4 Countries identified for further collecting per sweetpotato [*Ipomoea batatas* (L.) Lam.] crop wild relative.

| Taxon | Priority | Countries identified for further collecting |
| --- | --- | --- |
| *I. batatas* | HPS | Belize, Bolivia, Brazil, Colombia, Costa Rica, Ecuador, El Salvador, French Guiana, Guatemala, Guyana, Honduras, Mexico, Nicaragua, Panama, Peru, Suriname, Venezuela |
| *I. cordatotriloba* | HPS | Argentina, Bolivia, Brazil, Mexico, Paraguay, USA |
| *I. cynanchifolia* | HPS | Bolivia, Brazil |
| *I. grandifolia* | MPS | Argentina, Bolivia, Brazil, Paraguay, Uruguay |
| *I. lacunosa* | HPS | USA |
| *I. leucantha* | HPS | Mexico, USA |
| *I. littoralis* | HPS | Australia, Fiji, French Polynesia, Guam, India, Indonesia, Kiribati, Madagascar, Malaysia, Mauritius, Micronesia, N. Mariana Islands, New Caledonia, Papua New Guinea, Philippines, Seychelles, Sri Lanka, Taiwan, Thailand, Timor-Leste, USA |
| *I. ramosissima* | HPS | Belize, Bolivia, Brazil, Colombia, Costa Rica, Ecuador, El Salvador, Guatemala, Honduras, Mexico, Nicaragua, Panama, Paraguay, Peru, Venezuela |
| *I. splendor-sylvae* | HPS | Belize, Costa Rica, El Salvador, Guatemala, Honduras, Mexico, Nicaragua, Panama |
| *I. tabascana* | LPS | Mexico |
| *I. tenuissima* | HPS | Cuba, Dominican Republic, Haiti, Puerto Rico, USA |
| *I. tiliacea* | HPS | Bahamas, Belize, Brazil, Colombia, Costa Rica, Cuba, Dominican Republic, El Salvador, French Guiana, Grenada, Guatemala, Guyana, Haiti, Honduras, Jamaica, Mexico, Nicaragua, Panama, Puerto Rico, Sao Tome and Principe, St. Vincent and the Grenadines, Suriname, USA, Venezuela |
| *I. trifida* | MPS | Belize, Colombia, Costa Rica, Cuba, Ecuador, El Salvador, Guatemala, Honduras, Mexico, Nicaragua, Panama, USA, Venezuela |
| *I. triloba* | HPS | Bahamas, Belize, Colombia, Costa Rica, Cuba, Dominican Republic, Ecuador, El Salvador, Guatemala, Haiti, Honduras, Jamaica, Mexico, Nicaragua, Panama, Peru, USA, Venezuela |
